# Biomarkers of the Endocannabinoid System in Substance Use Disorders

**DOI:** 10.3390/biom12030396

**Published:** 2022-03-03

**Authors:** Francisco Navarrete, María S. García-Gutiérrez, Ani Gasparyan, Daniela Navarro, Francisco López-Picón, Álvaro Morcuende, Teresa Femenía, Jorge Manzanares

**Affiliations:** 1 Instituto de Neurociencias, Universidad Miguel Hernández-CSIC, Avda. de Ramón y Cajal s/n, San Juan de Alicante, 03550 Alicante, Spain; fnavarrete@umh.es (F.N.); maria.ggutierrez@umh.es (M.S.G.-G.); agasparyan@umh.es (A.G.); dnavarro@umh.es (D.N.); amorcuende@umh.es (Á.M.); tfemenia@umh.es (T.F.); 2 Departamento de Medicina Clínica, Instituto de Investigación Sanitaria y Biomédica de Alicante (ISABIAL), Universidad Miguel Hernández, 03010 Alicante, Spain; 3 Redes de Investigación Cooperativa Orientada a Resultados en Salud (RICORS), Red de Investigación en Atención Primaria de Adicciones (RIAPAd), Instituto de Salud Carlos III, MICINN and FEDER, 28029 Madrid, Spain; 4 PET Preclinical Imaging Laboratory, Turku PET Centre, University of Turku, 20520 Turku, Finland; francisco.lopez@utu.fi

**Keywords:** endocannabinoid system, biomarker, substance use disorder, rodents, human, polymorphism, molecular biology, neuroimaging

## Abstract

Despite substance use disorders (SUD) being one of the leading causes of disability and mortality globally, available therapeutic approaches remain ineffective. The difficulty in accurately characterizing the neurobiological mechanisms involved with a purely qualitative diagnosis is an obstacle to improving the classification and treatment of SUD. In this regard, identifying central and peripheral biomarkers is essential to diagnosing the severity of drug dependence, monitoring therapeutic efficacy, predicting treatment response, and enhancing the development of safer and more effective pharmacological tools. In recent years, the crucial role that the endocannabinoid system (ECS) plays in regulating the reinforcing and motivational properties of drugs of abuse has been described. This has led to studies characterizing ECS alterations after exposure to various substances to identify biomarkers with potential diagnostic, prognostic, or therapeutic utility. This review aims to compile the primary evidence available from rodent and clinical studies on how the ECS components are modified in the context of different substance-related disorders, gathering data from genetic, molecular, functional, and neuroimaging experimental approaches. Finally, this report concludes that additional translational research is needed to further characterize the modifications of the ECS in the context of SUD, and their potential usefulness in the necessary search for biomarkers.

## 1. Introduction

According to the latest epidemiological data, the estimated number of past-year users of any drug globally stands at 275 million people, increasing by 22 percent between 2010 and 2019. From this population, almost 36.3 million (13%) suffer from diagnosed substance use disorders (SUD) [1]. SUD is a chronic, relapsing clinical condition characterized by compulsive drug-seeking and use despite harmful consequences, constituting one of the leading causes of disability and mortality. Indeed, deaths directly related to SUD amounted to 167,750 in 2015, representing a 60% increase over the previous figure of 2000. In short, opioids produce the highest morbidity and mortality among the drugs consumed, alcohol constitutes the most consumed legal drug globally, and cannabis is the illegal drug with the highest number of users [1,2].

Unfortunately, despite the devastating worldwide impact of SUD, available pharmacological treatments remain insufficiently effective for most people, probably due to the difficulties of characterizing and elucidating the underlying neurobiological mechanisms. While clinically useful, commonly used psychosocial and symptomatic criteria are not enough to accurately capture the vast heterogeneity of SUD, depending on factors such as genetics, age, gender, polydrug use, or psychiatric comorbidities. Furthermore, the current diagnosis of SUD relies on several qualitative outcome measures. For this reason, identifying central and peripheral biomarkers applying a multidisciplinary approach is an urgent need to diagnose the severity of drug dependence, monitor therapeutic efficacy, predict treatment response, or enhance the development of safer and more effective therapeutics for SUD [3,4,5,6].

In recent years, compelling advances have been acquired in understanding how addictive drugs affect the brain initially, particularly in the brain’s reward system, and induce longer-lasting neuroadaptive changes after repeated exposure, leading to the compulsive seeing and taking of drugs that define addiction [7]. The acute administration of drugs of abuse enhances the activity of the mesolimbic dopamine (DA) system [8], increasing DA release in the nucleus accumbens (NAcc) shell through the stimulation of DA neurons from the ventral tegmental area (VTA). This neurochemical response has been related to the rewarding effect [9], producing a hedonic experience that is crucial for the initiation and maintenance of drug consumption [10]. Repeated drug exposure causes adaptive changes in the circuitry of the extended amygdala, resulting in enhanced reactivity to stress [11] and the emergence of negative emotions [12]. Furthermore, chronic drug consumption also leads to profound impairments in decision-making that are closely related to functional and morphological changes occurring in the prefrontal cortex (PFC) [13]. The PFC and other frontal regions play a crucial role in executive processes, including self-regulation, behavioral control, flexibility, and threat conditioning [14,15,16,17]. A down-regulation of DA signaling occurs in the PFC and their associated circuits after repeated drug exposure. These changes are linked to neuroplastic and morphological changes in the PFC glutamatergic neurons [18]. In addition, it is relevant to note that, apart from disturbances mainly in dopaminergic and excitatory neurotransmission systems, the phenomena encompassing drug addiction are also associated with neuroinflammatory processes that have recently received particular attention [19], and are related to the activation of metabolic systems, such as the tryptophan-kynurenine pathway [20,21,22]. Overall, these alterations weaken the inhibitory control that cortical areas exert on the mesolimbic system, impairing the capability to control the decisions related to drug consumption, finally leading to the loss of control, compulsive drug use and relapse that characterize addictive disorders.

**Figure 1:**
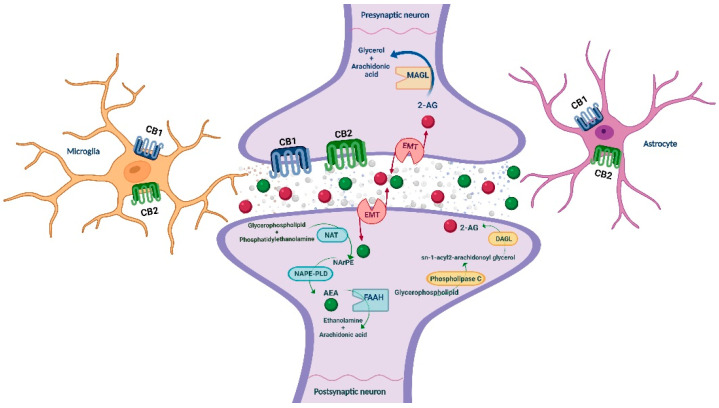
Schematic representation of the main ECS components, including the metabolizing routes of the eCBs. AEA: anandamide; CB1/CB2: cannabinoid receptors 1 and 2; DAGL: diacylglycerol lipase; EMT: endocannabinoid membrane transporter; FAAH: fatty acid amide hydrolase; MAGL: monoacylglycerol lipase; NAPE-PLD: N-acylphosphatidylethanolamine specific phospholipase D; NArPE: N-arachidonoyl phosphatidylethanolamine; NAT: N-acyl transferase; 2-AG: 2-arachidonoylglycerol. Image created with BioRender.

The endocannabinoid system (ECS) has received substantial attention, and accumulated evidence points out its crucial role in the neuromodulation of the rewarding and neurophysiological actions of drugs of abuse [23,24]. ECS is a ubiquitous lipid signaling system distributed throughout the organism that participates in multiple intracellular signaling pathways [25,26]. It regulates several physiological functions and mediates the crosstalk between different neurotransmitter systems, therefore representing a key player in controlling behavioral responses [27,28]. In short, cannabinoid receptors (CB1R and CB2R), endogenous ligands or endocannabinoids (eCBs; anandamide (AEA) and 2-arachidonoylglycerol (2-AG)), and the major enzymes responsible for the synthesis (N-acylphosphatidylethanolamine specific phospholipase D (NAPE-PLD) and diacylglycerol lipase (DAGL)) and degradation (fatty acid amide hydrolase (FAAH) and monoacylglycerol lipase (MAGL)) of eCBs are the main components of the ECS, present in the central and peripheral nervous system [27,29] (see Figure 1), and in many other peripheral tissues regulating distinct functions [30].

The connection between the ECS and drug addiction emerges from the very well-known rewarding effects and abuse potential of cannabis preparations. Notably, the phytocannabinoid delta-9-tetrahydrocannabinol (THC) accounts for typical cannabis effects. In addition, ECS components are expressed in the brain regions that make up the mesocorticolimbic pathway, including the VTA, NAcc, or PFC. Notably, the dopaminergic neurons of this brain reward circuitry are controlled by excitatory and inhibitory inputs that are, in turn, modulated by the ECS [31,32,33]. 

Thus, identifying drug-induced changes in the targets comprising this neuromodulatory system has attracted increasing attention in recent years to discover new biomarkers with diagnostic, prognostic, or therapeutic potential. The present review compiles the primary evidence on the genetic (polymorphisms), molecular, functional and neuroimaging alterations in the components of the ECS (receptors, endocannabinoids and enzymes) that occur as a consequence of exposure to different drugs of abuse (alcohol, cannabis, opioids, stimulants, nicotine, and hallucinogens), from a translational approach that integrates clinical and animal studies. 

## 2. Methods

The literature search for this narrative review was performed in the Medline database (PubMed) employing medical subject headings (MeSH). Specific keywords were employed according to the substance-related disorders included in the review: Alcohol (“ethanol” [MeSH]), cannabis [MeSH], analgesics, opioid [MeSH], N-Methyl-3,4-methylenedioxyamphetamine [MeSH], methamphetamine [MeSH], cocaine [MeSH], nicotine [MeSH], tobacco [MeSH], hallucinogens [MeSH]. These terms were combined with “cannabinoids” [MeSH] by the Boolean operator “AND.” All the authors critically analyzed all the results for each search to decide the selection of each reference according to the adequacy of its content with the subject matter of the study. No PubMed filters were applied to maximize the selection of all the available and appropriate information. All original articles, systematic reviews, or meta-analyses on identifying ECS components alterations in drug addiction were accepted. Those articles not related to the topic of interest, not written in English, or to which access was not possible were discarded. 

## 3. Endocannabinoid Components as Potential Biomarkers in SUD

This section aims to gather evidence about how the ECS is impaired due to acute or chronic drug consumption or to specific stages of drug-related disorders (alcohol, cannabis, opioids, stimulants, tobacco, and hallucinogens), mainly intoxication, withdrawal, dishabituation, or relapse. For this purpose, a translational approach has been applied to combine animal and human studies providing relevant information from a multidisciplinary point of view that includes genomics, epigenetics, genetics, proteomics, or neuroimaging.

### 3.1. Alcohol-Related Disorders

Alcohol use disorder (AUD) is one of the most common addictive disorders, with significant health and socioeconomic impacts. The search for adequate biomarkers allowing the development of preventive and therapeutic strategies is a significant challenge to improve the clinical management of alcoholic patients. In this regard, ECS components have attracted great interest because of their potential to serve as biomarkers in AUD. This section gathers the most recent studies developed to detect and describe changes in the ECS in patients with AUD and different animal models of ethanol exposure, providing valuable information about its potential diagnostic, prognostic, or therapeutic usefulness (Table 1).

#### 3.1.1. Gene Polymorphisms of ECS Components

Promising clinical results have been obtained by evaluating the involvement of different single-nucleotide polymorphisms (SNPs) of genes encoding distinct cannabinoid components in AUD. Considering the close relationship between the ECS and dopaminergic system, Hoenicka et al. aimed to analyze genetic factors, underlying the dual pathology of AUD and antisocial personality disorder. The authors evaluated gene variants involved in both systems, analyzing SNPs near the D2 dopamine receptor gene (D2). More specifically, a 10-repeat allele of a variable number tandem repeats (VNTR) of the *SLC6A3* gene, the C385A *FAAH* SNP and the 3′-UTR microsatellites of the *CNR1* gene were analyzed. In a sample of 137 alcoholic patients, a close correlation was found in *CNR1* and *FAAH* genes. This result suggests the interaction of the dopaminergic and ECS systems in developing the comorbidity of alcohol misuse and antisocial behavior [34]. However, the interaction between *CNR1* (rs2023239) and the D4 dopamine receptor gene (*DRD4*) did not provide similar results regarding the interconnections between the dopaminergic and cannabinoid systems. The *CNR1* C allele group presented more increased alcohol craving than the T allele group, but no correlations were found between *DRD4* and *CNR1* gene variants [35]. 

Seven SNPs were significantly associated with Alcohol Use Disorder Identification Test (AUDIT) scores in a European adolescent population of alcohol consumers. Two of them, *CNR1* (rs9343525) and monoacylglycerol lipase (*MGLL*; rs507961) genes, remain significant after statistical corrections for multiple covariables [36]. When evaluating *FAAH* and *MGLL* genes SNPs in an alcoholic population of Japanese patients, despite covering most regions of the genes encoding these endocannabinoid metabolic enzymes, no associations were observed with the increased susceptibility to develop alcoholism [37]. However, the polymorphism Q63R of the *CNR2 gene* was associated with high ethanol consumption in another mixed sample (including males and females) of the Japanese population [38]. 

Two polymorphisms of *CNR1*, rs6454674 (SNP3) and rs806368 (SNP8), have been strongly associated with both alcohol dependence and drug dependence. Interestingly, the interaction between both SNPs significantly increased the risk of developing drug or alcohol dependence [39]. The influence of these two allelic variants of *CNR1* (rs6454674 and rs806368) with an additional one (rs1049353) was evaluated in another study developed by Marcos and colleagues [40]. The authors analyzed single markers and haplotypes and conducted interaction analyses, concluding that the haplotype TGT (corresponding to rs6454674-rs1049353-rs806368) decreases the susceptibility to develop alcohol dependence. However, an interaction was found between the G allele of rs6454674 SNP and the C allele of rs806368 SNP, supporting the involvement of the ECS in alcohol dependence [40]. The polymorphism rs1049353 (1359 G/A, Thr453Thr) was also evaluated in a Caucasian population, comparing the frequency of its presentation in alcohol and control subjects. In this study, the authors showed that the homozygous genotype *CNR1* 1359 A/A of the polymorphism increased the vulnerability to develop alcohol withdrawal-induced delirium [41]. Thus, identifying this genetic variant could be an exciting strategy in alcoholic patients to prevent the development of delirium. 

Previous studies pointed out that the A or G allele of a *CNR1* polymorphism could not be associated with a history of alcohol withdrawal-induced seizures [42]. Nevertheless, post-mortem studies showed a strong association between the C allele of the *CNR1* rs2023239 polymorphism, with increased CB1R binding in the prefrontal cortex, alcohol cue-elicited brain activation, and subjective alcohol reward. Thus, patients with the C allele are more susceptible to changes in the mesocorticolimbic circuitry, and more prone to developing alcohol dependence [43]. 

The SNP of the *FAAH* gene, C385A (*FAAH* Pro129Thr, rs324420), is associated with a decreased enzymatic activity of FAAH, resulting in increased anandamide levels and activity. This *FAAH* SNP has been related to alterations in alcohol consumption. In a study developed by Bühler and colleagues, authors evaluated the involvement of 10 SNPs of different targets, two of them corresponding to the *FAAH* gene. The positive rating of alcohol-related pictures was associated with the homozygous CC C38A SNP genotype, indicating that this SNP is a candidate for screening patients with a higher risk of alcohol-related problems, such as sleep disturbances [44,45]. This correlation appears to be different depending on the ethnic factors. Indeed, the Thr129 allele frequency is higher in European American participants with current alcohol dependence than in non-dependent controls. Thr129 carriers reported a median of 10 fewer abstinent days and 13 more binge drinking days than controls. Nevertheless, there were no significant differences between Thr129 allele frequency in African American participants in alcoholic patients and controls [46]. 

The minor allele (A) of the SNP C385A has been associated with reduced FAAH enzyme activity and increased risk for substance use disorders in adults. However, its implication for young people still has to be elucidated. For this purpose, Best and colleagues determined the *FAAH* C385A genotype in a group of heavy-drinking youths. Authors showed that people with the *FAAH* minor allele (AC or AA genotype) present significantly more drinking days, heavy episodic drinking, and higher alcohol-related problems and consumption patterns [47]. These findings suggested that the reduced endocannabinoid metabolism induced by the SNP affecting the *FAAH* gene may be related to heavier alcohol use in the young population before the onset of chronic drinking problems. Thus, identifying this SNP may be a potential marker of possible development of alcohol dependence, allowing the implementation of preventive strategies. 

Previous results in humans were confirmed in a preclinical study using genetic knock-in mice containing the human *FAAH* SNP C385A. These mutant *FAAH* (A/A) mice developed a greater alcohol intake and preference in the drinking-in-the-dark paradigm (DID), supporting the idea about the involvement of this SNP in alcohol dependence [48]. 

#### 3.1.2. Gene and Protein Function/Expression Changes of ECS Components

The first evidence suggesting the involvement of ECS in the regulation of ethanol effects was that acute or chronic ethanol exposure modifies the gene expression of the CB1R. Acute ethanol exposure induced neuroplastic alterations in the *CNR1* gene expression in different brain areas such as caudate-putamen (CPu), central amygdala (CeA), and ventromedial hypothalamic nucleus [49]. These results were extended by Ortiz and colleagues, evaluating the effect of chronic ethanol exposure on *CNR1* gene expression. In this study, the consumption of ethanol for a 52-day period reduced the gene expression of *CNR1* in the CPu, the ventromedial nucleus of the hypothalamus, CA1 and CA2 fields of the hippocampus (Hipp), and increased gene levels in the dental gyrus in rats [50]. Furthermore, other authors showed that chronic ethanol vapor exposure increases *MGLL* and *DAGLB* mRNA and MAGL protein levels. These changes were accompanied by a significant decrease in *CNR1* mRNA and CB1R protein levels in alcohol-exposed rats [51]. The implication of *CNR1* genes in alcohol intake was also evaluated in a study using the CRISP/CAS9 method. Hay and colleagues disrupted a highly conserved regulatory sequence in the CNR1 gene in this study. This procedure reduced *CNR1* gene expression, causing a subsequent reduction in alcohol intake in the two-bottle choice paradigm, supporting the involvement of ECS in alcohol dependence [52].

The alcohol intake in rats exposed to a protocol of maternal separation during its postnatal period correlated with the increased protein expression of CB1R in the ventral striatum, and decreased in the frontal cortex [53]. These findings were similar to those obtained by Hansson and colleagues regarding FAAH levels in alcohol-preferring rats. In this study, the increased preference strongly correlated with decreased expression of FAAH in the prefrontal cortex (PFC), inducing reduced enzyme activity in this brain region. These results suggest an overactive endocannabinoid transmission of alcohol-preferring rats and a compensatory down-regulation of CB1R signaling [54]. On the other hand, mice with high alcohol preference showed reduced gene expression of *CNR2* in the ventral midbrain, supporting the idea of the involvement of this gene in alcohol consumption [38].

ECS changes were also reported in rodents exposed to alcohol withdrawal paradigms. Acute alcohol withdrawal reduced AEA content in the basolateral amygdala (BLA) and 2-AG concentrations in the PFC. Interestingly, authors observed these alterations in male rats but not in females, suggesting sex-dependent changes in this animal model [55]. In addition, acute ethanol withdrawal was also associated with *FAAH*, *MGLL*, *CNR1*, *CNR2* and *GPR55* gene expression reduction. These changes were more pronounced than those obtained after intermittent alcohol exposure [56]. The intermittent alcohol exposure increased the mRNA levels of the enzyme of synthesis of AEA and 2-AG in the PFC and reduced it in the amygdala (Amy). On the other hand, this type of alcohol exposure was associated with decreased mRNA levels of *CNR1* and *CNR2* in the striatum [57]. 

Ventral striatum post-mortem samples of alcohol-dependent patients were analyzed to evaluate changes in the CB1R protein levels, enzymatic activity, and protein levels of FAAH. The results revealed reduced CB1R levels, CB1R-mediated signaling, and FAAH levels compared with the control group. Authors suggested that alterations in FAAH activity modified AEA concentrations, CB1R protein levels, and receptor activity [58]. However, further studies are required to explore the relationship between CB1R in the ventral striatum of alcoholic patients and the development of alcohol dependence. CB1R, CB2R and GPR55 gene and protein levels were also evaluated using human monocyte-derived dendritic cells from alcohol users. Gene expression studies revealed an increase in *CNR2* and *GPR55* gene expressions without changes in *CNR1*. Interestingly, both receptors increased after in vitro treatment, with different concentrations of ethanol in cell cultures [59]. 

#### 3.1.3. Neuroimaging of ECS Components

The first human studies showing the critical role of the endocannabinoid system (ECS) were performed using the CB1R binding radiotracer [^11^C]OMAR [61]. This study was conducted in a relatively limited group of men (8 healthy controls and 8 with alcohol dependence, 4 weeks after the last drink). Interestingly, the volume of distribution (*V*_T_) of [^11^C]OMAR in patients with alcohol dependence was approximately 20% (*p* = 0.023) higher than in healthy controls in the Amy, Hipp, putamen, insula, anterior and posterior cingulate cortices and orbitofrontal cortex (OFC). Age, body mass index or smoking status did not influence the outcome. In a similar but amplified study with the radiotracer [^18^F]FMPEP-*d*_2_, Hirvonen et al. scanned 18 male in-patients with alcohol dependence twice, within 3–7 days of admission from ongoing drinking, and after 2–4 weeks of supervised abstinence, and compared them to 19 healthy males. On the first scan, CB1R binding was 20–30% lower in patients with alcohol dependence than in control subjects in all brain regions, and was negatively correlated with years of alcohol abuse. Indeed, this result was in agreement with data from the previous study. In the second scan after 2–4 weeks of abstinence, the CB1R levels did not show significant recovery in those patients, indicating the long-term effects of alcohol dependence and the involvement of CB1R [62]. In a posterior study, Ceccarini et al. [63] investigated the changes in CB1R availability after acute and chronic alcohol abuse. The 20 healthy subjects that received an intravenous ethanol injection to study the acute effects showed a global brain increase in CB1R availability (+15.8%) measured with [^18^F]MK-9470. In contrast, the chronic drinking patients showed a global decrease (−16.1%). After 1 month of abstinence, the decreased availability of CB1R persisted, according to Hirvonen et al., 2013.

Studies in rodents using PET also investigated the effect of alcohol in the ECS. Ceccarini et al. showed that acute alcohol administration in male rats increased [^18^F]MK-9470 binding to CB1R in the NAcc, while chronic ethanol consumption decreased [^18^F]MK-9470 in the Hipp and CPu. In contrast to human studies, the [18F]MK-9470 levels recovered the baseline levels in rats and increased after 7 and 14 days of abstinence [60].

In addition to radiotracers targeting CB1R and CB2R, others targeting the two main hydrolases that metabolize AEA and 2-AG (FAAH and MAGL, respectively) are under development. [^11^C]CURB was the first PET radiotracer developed to image FAAH, and evaluated first in animals [65], and later in humans [66]. Best et al. studied brain FAAH levels in AUD patients 3–7 days and 2–4 weeks after abstinence using [11C]CURB. They found that brain FAAH levels were lower in alcoholic patients than in controls during early abstinence, while there were no differences at 2–4 weeks [64]. 

#### 3.1.4. Concluding Remarks—ECS and Alcohol-Related Disorders

Studies developed to date showed the strong involvement of ECS in regulating alcohol effects in humans and animal models. Some relevant conclusions can be drawn from the available evidence: (1) specific CNR1 polymorphisms have been widely associated with AUD; (2) FAAH C385A rs324420 polymorphism seems to be related to increased risk of alcohol misuse and addiction; and (3) chronic alcohol exposure commonly decreases FAAH (gene expression and binding) associated with an increase in eCBs levels and reduced CB1R (gene expression and binding) in several brain regions. Further studies are required to explore, discover, and characterize specific ECS biomarkers with diagnostic or therapeutic potential in alcohol-related disorders.

### 3.2. Cannabis-Related Disorders

Much research on cannabis has focused on studying dynamic changes in the gene and protein expression of key targets within the ECS, induced by THC or cannabinoids, mainly in rodents, and to a lesser extent, in humans. This review summarizes the most promising results, revealing significant alterations that may serve as potential biomarkers for cannabis use disorders (CUD) (Table 2).

#### 3.2.1. Gene Polymorphisms of ECS Components

In humans, genetic variants of different critical elements of the ECS have been proposed as a risk factor for cannabis dependence, withdrawal, craving, and cue-elicited brain activation. 

A significant correlation was found between the *CNR1* SNPs rs1049353, rs806368, and rs806380 and CUD [67,69,70]. The polymorphism *CNR1* rs2023239, allele G, is associated with more significant withdrawal and craving for cannabis after short-term abstinence, greater craving after cannabis cue exposure [71], and more significant brain response to cannabis cue in several areas, including the OFC, inferior frontal gyrus (IFG), and anterior cingulate gyrus (ACG) [72]. It has been found that allele G affects *CNR1* mRNA expression; in human postmortem brain tissues, a reduction of *CNR1* gene expression was detected in the PFC. Moreover, cannabis users’ carriers of the G allele showed smaller hippocampal volume, suggesting that cannabis exposure may interact with CB1R altered by the polymorphism, in such a way that reduces hippocampal volume [73]. 

Colizzi et al. performed a functional MRI study in cannabis users, demonstrating that G carriers of the *CNR1* SNP rs1406977 had superior functional connectivity in the left ventrolateral PFC, and reduced working memory accuracy during the 2-back task. They concluded that the adverse effects of cannabis use are enhanced on a specific genetic background related to *CNR1* variations [74]. A longitudinal structural MRI study on regular users of cannabis found an association between reduced volume in the anterior cingulum and *CNR1* SNPs 1049353 and rs806368. Authors proposed an association between the level of cannabis exposure, the decreased volume of the brain mentioned above the area, and *CNR1* haplotypes variation [68]. 

Similarly, Ho et al. found a correlation between the *CNR1* SNP rs12720071 and smaller parietal white matter volume in schizophrenic patients with CUD [75]. Interestingly, a recent publication linked the polymorphisms *CNR1* rs12720071 (G-allele-carriers), the mitogen-activated protein kinase 14 (MAPK14) rs12199654 (A-allele carriers), and smaller cerebral and lobar white matter volumes in schizophrenic patients with heavy marijuana use [76]. 

In the case of the CB2R, the SNP rs2501431 (*CNR2*) has been associated with CUD [77]. Recently, the SNPs rs12744386 and rs35761398 (*CNR2*) have been linked with a high risk for developing schizophrenia in patients with CUD [78]. 

The human polymorphism *FAAH* C385A (rs324420) is one of the most explored SNPs. This polymorphism results in a mutant form of FAAH, with reduced expression and cellular stability [79,110]. The A allele has been associated with street drug use [79] and allele C with an increased risk for cannabis dependence progression, although with inconsistent results. On the one hand, allele A has been linked with increased ventral striatum reactivity, brain area closely related with reward, higher impulsivity [80], and more significant bias to appetite stimuli [81]. In other studies, the allele C was associated with the increased activity of brain reward areas in regular marijuana users [72].

Similarly, opposite results were found in the role of alleles A and C in the progression from sporadic cannabis users to cannabis dependents. Some authors found a link between allele A and an increased risk for CUD [79,82,111,112], while others revealed that allele C confers more risk for THC dependence [71,78,83]. Recently, allele C has been associated with craving during marijuana abstinence [71]. 

Recent research has deeply explored the role of C385A polymorphism in cannabis dependence by using a knock-in mouse model (FAAHC/A) that biologically recapitulates the human polymorphism. Interestingly, the authors found a modified *CNR1* gene expression with higher levels at GABAergic terminals and lower levels at glutamatergic terminals in the VTA, which may affect the development of the mesolimbic reward pathway. This study supports the fact that this polymorphism C/A contributes to a preference for THC in adolescent female mice that persists into adulthood compared to female FAAHC/C, which find THC aversive [99]. Furthermore, the SNP rs604300 within the *MGLL* has been associated with stress adaptation related to cannabis dependence [84]. A recent study showed changes in white matter bundles innervating posterior cingulate and parietal cortex, basal ganglia, and temporal cortex in patients with cannabis dependence, and a significantly lower gray matter thickness and density in the precuneus. These brain structural alterations seemed to be significantly associated with regional differences in MAGL expression [102].

#### 3.2.2. Gene and Protein Function/Expression Changes of ECS Components

Most of the results come from studies in rodents focused on identifying the effects of chronic cannabinoid agonists administration to clarify the mechanisms underlying cannabis dependence, craving, and, more recently, cannabinoid withdrawal. 

Chronic cannabinoid administration induced a dynamic and time-dependent reduction of *CNR1* mRNA levels in the different brain regions of rodents, such as the CPu. During the first days (5–7 days) of chronic administration, there was no alteration in *CNR1* gene expression, but it was markedly reduced at 11 days of administration. These changes occur parallel to motor tolerance, supporting the involvement of *CNR1* mRNA reduction in the development of cannabis tolerance [85,86]. However, in an additional study, chronic THC administration during 5 days induced an opposite effect, since *CNR1* mRNA levels were increased in the striatum of male Wistar rats [87]. The discrepancy in the results may be due to the different cannabinoid agonists used (CP-55,940 and THC), the pattern of administration (doses, duration), and the strain of rodents used (rats). 

Male rats perinatally exposed to chronic THC showed reduced *CNR1* gene expression in the PFC and lower 2-AG brain concentrations; changes persist until adulthood. The alterations in *CNR1* gene expression have been associated with changes in dopamine D2 receptor (*DRD2*) gene expression and, even more critically, with a significant hypomethylation at the *DRD2* regulatory region [113]. Altogether, these data emphasize the involvement of CB1R in cannabis dependence and its close interaction with the dopamine reward system [114,115].

In humans, increased the CB1R gene expression and hypomethylation of the promotor were detected in the peripheral blood cells of THC-dependent patients [100]. More importantly, *CNR1* mRNA levels correlate with a lower score on the Satisfaction With Life Scale (SWLS) and with craving and cannabis dependence, results in agreement with previous animal studies showing a relationship between craving and *CNR1* gene expression [116]. 

In mice models of cannabinoid withdrawal, *CNR1* gene expression was increased in the NAcc, the ventromedial hypothalamic nucleus, CeA, and CA1 field of the Hipp [89,90,91,117]. This up-regulation may be due to a compensatory neuroadaptive response to reducing CB1R found after repeated treatment with a cannabinoid receptor agonist [86,118]. Besides, CB2R gene expression has been down-regulated in the NAcc of C57BL/6J mice exposed to cannabinoid withdrawal [91].

Autoradiographic studies revealed that the chronic administration of THC or CB1R agonists, such as the CP-55,940, significantly reduced CB1R binding and led to tolerance in most responses (e.g., motor tolerance) [92,93,96,118,119,120]. The down-regulation and desensitization of CB1R are time-dependent and region-specific, and occur prior to gene expression alterations [94]. Thus, the reduction of CB1R binding may represent the first step in the biochemical mechanisms underlying tolerance to cannabinoids [95,121].

In rats, the acute administration of THC induced an upward shift of AEA concentrations in the NAcc, though there was still a trend towards increased levels after complete chronic treatment [88]. Previous studies found increased concentrations of AEA in limbic forebrain areas after chronic THC administration [97,98]. Thus, cannabinoid tolerance is accompanied by region-dependent changes in the concentrations of AEA and 2-AG [97,98]. In healthy volunteers, a single oral dose of THC increased circulating concentrations of AEA, 2-AG, and other endocannabinoids 2 and 3 h after administration compared with placebo [101]. 

#### 3.2.3. Neuroimaging of ECS Components

The effects of the chronic use of cannabis on ECS brain components were investigated using PET imaging. Hirvonen et al. investigated the changes in CB1R availability with [^18^F]FMPEP-*d*_2_ in 30 chronic daily cannabis smokers and 28 control subjects. They imaged the chronic cannabis smokers, the day after admission, and approximately 4 weeks after abstinence. At baseline, the *V*_T_ of [^18^F]FMPEP-*d*_2_ was lower in cannabis smokers than in control subjects in neocortex and limbic areas. After the abstinence period, the *V*_T_ of [^18^F]FMPEP-*d*_2_ showed significant increases in regions, with reduced *V*_T_ at baseline [103]. Ceccarini et al. imaged 10 chronic cannabis users and 10 healthy controls using [^18^F]MK-9470 to measure CB1R availability. The results showed a global decrease in CB1R receptor availability (−11.7%), the most significant changes in the temporal lobe, anterior and posterior cingulate cortex and NAcc [104]. Bhattacharyya et al. investigated the effect of THC on anxiety and amygdala response in humans using the CB1R binding radiotracer [^11^C]MePPEP. The THC treatment-induced anxiety modulated Amy activation and increased the availability of CB1R [105]. D’Souza et al. investigate the time course of changes in CB1R availability following short and intermediate-term abstinence in a group (n = 11) of cannabis-dependent subjects, imaging them with [^11^C]OMAR just before starting abstinence and 2 and 28 days after. *V*_T_ was lower in cannabis-dependent subjects than the healthy controls at baseline. However, this difference was no longer evident after 2 or 28 days of abstinence [106].

Changes in FAAH were investigated in cannabis use patients. Boileau et al. studied the FAAH alterations using [^11^C]CURB in a small group of chronic cannabis users (n = 10) during early abstinence, compared to a group of healthy controls (n = 22). They showed decreased [^11^C]CURB binding in the chronic cannabis users from −20% (Amy and cingulate) to −14% (Hipp), and in most of the cortical areas studied [107]. In a more recent study from the same researchers, the results showed lower [^11^C]CURB binding among a group (n = 14) of young chronic cannabis users (23 ± 5 years of age) compared to healthy controls during early abstinence throughout the whole neocortex and striatum, Amy, Hipp, thalamus and cerebellum [108]. Another study using [^11^C]OMAR studied the changes in CB1R availability in women with CUD (n = 10), who displayed significantly lower *V*_T_ than healthy female controls (n = 10) in the Hipp, Amy, cingulate, and insula, similar to previous studies in men [109]. Interestingly, a recent review on psychiatric disorders also evaluated PET studies in CUD and AUD [122].

The effects of ECS genetic polymorphisms in the binding of radiotracers were described in recent studies. Boileau et al. studied FAAH changes using [^11^C]CURB in a small group of chronic cannabis users, showing that the brain binding of the [^11^C]CURB is dependent on the genetic *FAAH* polymorphism rs324420 (C385A) [107]. 

#### 3.2.4. Concluding Remarks—ECS and Cannabis-Related Disorders

The evidence suggests a strong involvement of ECS in regulating cannabis effects in humans and animal models. Some relevant conclusions can be drawn from the available evidence: (1) specific CNR1 polymorphisms have been associated with significant cannabis withdrawal syndrome and craving; (2) the consequences of *FAAH* C385A rs324420 polymorphism on cannabis effects and the development of cannabis dependence vary according to the carrying allele (C or A); and (3) cannabis chronic exposure induces a down-regulation of CB1R (gene expression and binding) that is normalized or even increased during withdrawal. Further studies are required to explore, discover, and characterize specific ECS biomarkers with diagnostic or therapeutic potential in cannabis-related disorders. 

### 3.3. Opioid-Related Disorders

The crosstalk between the endogenous opioid and cannabinoid systems has a pivotal role in regulating the rewarding and neurophysiological actions of several abuse drugs, including opioids (i.e., morphine, heroin) [123,124,125]. This section contains the available data from animal and clinical studies about the genetic, molecular and neuroimaging modifications in ECS components related to opioid addiction (Table 3).

#### 3.3.1. Gene Polymorphisms of ECS Components

There is very scarce information about ECS polymorphisms related to opioid addiction. One study aimed to genotype 6 polymorphisms of the *CNR1* gene and the 385C>A SNP of the *FAAH* gene in former heroin addicts and their corresponding controls distinguishing between ethnicities (Caucasians, Hispanics, African Americans and Asians). Long repeats of the triplet polymorphism of *CNR1* 18087-18131(TAA)_8–17_ were significantly associated with vulnerability to develop heroin addiction. Conversely, the allele 1359A and the genotype 1359AA of *CNR1* were associated with a protective effect, particularly in Caucasians. However, no association was found with the missense substitution 385C>A in the *FAAH* gene [126]. Another work focused on evaluating the association of the *CNR1* SNP rs2023239 and the development of major depressive disorder (MDD) and/or suicidal behavior in opiate-dependent outpatients under stable methadone treatment. Interestingly, after regression analysis adjusting for covariables, the C allele of *CNR1* rs2023239 was closely related with a lower prevalence of lifetime MDD, but not with suicidal behavior. Although authors acknowledged that replication studies were necessary, these results were encouraging, since the early detection of opioid addicts at risk of major depression was crucial to better implementing psychiatric care in this population [127].

#### 3.3.2. Gene and Protein Function/Expression Changes of ECS Components

One of the first steps to elucidate the involvement of ECS in opioid addiction was the evaluation of the effects of acute or chronic morphine administration on the expression or function of distinct ECS components. Romero and colleagues published the first study showing changes in CB1R binding in different brain regions under basal ([^3^H]CP-55,940) or stimulated ([^35^S]GTPγS + WIN-55,212-2) conditions in Swiss mice exposed to morphine (5 days, twice daily, 8–45 mg/kg, s.c.). Although CB1R binding levels were very similar between morphine-dependent and control mice, a small but significant increase was found in the globus pallidus. In addition, the WIN-55,212-2-stimulated [^35^S]GTPγS binding to CB1R was significantly higher in the substantia nigra and central gray substance. These data suggested that morphine might alter the coupling to intracellular mechanisms rather than cannabinoid receptor binding [128]. 

Subsequent studies continued to evaluate the effects of repeated morphine exposure (6 days, twice daily, 10–100 mg/kg, i.p.) on CB1R binding and gene expression in different brain regions of Wistar rats. Morphine administration increased CB1R binding in medial CPu, septum and NAcc while decreasing it in the dental gyrus and BLA. Furthermore, *CNR1* mRNA levels measured by in situ hybridization were significantly reduced by morphine exposure in the CPu and cerebellum. In contrast, an up-regulation was found in the CA2 region of the Hipp and septum [129]. The authors of the previous study employed the same procedure of morphine exposure to further characterize the changes in eCBs and their receptors in different brain regions involved in drug reward. AEA levels were similar between morphine-dependent and control rats, while CB1R binding was reduced in the cerebral cortex and midbrain of rats exposed to morphine. Moreover, CB1R-stimulated [^35^S]GTPγS binding increased in the cerebral cortex and decreased in the brainstem. These alterations in ECS components suggested that its pharmacological manipulation might represent a novel tool to manage the negative consequences of opioid addiction [130]. 

Additional studies also aimed to fully characterize the alterations of ECS components in the reward system after sustained morphine treatment. Vigano et al. chronically treated Sprague–Dawley rats with morphine (4.5 days, twice daily, 5 mg/kg, s.c.) and evaluated CB1R binding, CB1R-stimulated [^35^S]GTPγS binding, and eCBs levels. Morphine-tolerant rats showed reduced CB1R binding in the cerebellum and Hipp, decreased CB1R function in the NAcc, and lower 2-AG levels in limbic regions such as NAcc and Hipp [131]. Furthermore, repeated morphine treatment (5 days, twice daily, 5 mg/kg, i.p.) in the same rat strain induced a reduction of MAGL protein and *CNR2* gene expression in the VTA, whereas AEA or 2-AG levels were unaltered in this brain region. However, the authors also explored the effects of a single morphine injection and obtained a significant decrease in AEA levels [132]. Indeed, Jin and colleagues evaluated the differences between the administration of morphine under acute (10 mg/kg, s.c.) or chronic (12 days, twice daily, 10 mg/kg, s.c.) conditions on CB1R in several brain areas of Wistar rats. Prolonged morphine administration significantly increased CB1R gene and protein expression in the cortex, cerebellum, and Hipp. Acute treatment did not modify CB1R protein levels in these regions, and decreased *CNR1* gene expression in the cerebellum. Interestingly, acute and particularly repeated morphine treatment induced an up-regulation of *CNR1* gene expression in peripheral blood mononuclear cells (PBMCs). This study provided relevant evidence about the effects of morphine on CB1R in the central nervous system and peripheral immune system cells, adding new clues to the involvement of the ECS in opioid addiction [133]. Moreover, the effects of acute (5 and 10 mg/kg, s.c.) and chronic (5 days, 10–40 mg/kg, s.c.) morphine treatment on AEA and 2-AG levels were also evaluated in the NAcc (shell subregion) of Wistar rats, obtaining an increase in AEA and a decrease in 2-AG [134].

The conditioned place preference (CPP) paradigm has been employed to analyze morphine-induced changes in ECS components in its different experimental phases (conditioning, extinction or withdrawal, and reinstatement). In Sprague–Dawley rats, 5 days of conditioning with morphine (10 mg/kg, s.c.) induced an up-regulation of *CNR2* gene expression in the cortex, spleen, and PBMCs, while decreasing in the brainstem. Notably, this study also included clinical samples from morphine abusers (n = 8) and their corresponding controls (n = 5). Despite the limited number of patients, higher levels of cannabinoid receptors gene expression were found in the PBMCs of subjects with at least 1 year of morphine overuse history compared with controls [135]. Later, Yuan et al. evaluated the effects of morphine withdrawal at different phases (acute, latent, and chronic) on CB1R protein expression after 5 days of conditioning (10 mg/kg, s.c.) in the CPP. Regardless of the phase, CB1R was elevated in the NAcc of rats undergoing morphine withdrawal, a result that was accompanied by an increase of CB1R-positive inhibitory terminals [136].

Moreover, short-term morphine withdrawal evaluated 72 h after the 7-days of conditioning (10 mg/kg, s.c.) in the CPP was associated with increased DAGLα in the NAcc [137]. These findings led authors to hypothesize the involvement of 2-AG on the CB1R-mediated modulation of inhibitory synaptic transmission in the NAcc under morphine withdrawal conditions, which may be one of the mechanisms associated with opioid relapse. Another study performed in C57BL/6J mice intended to characterize the gene expression alterations of ECS components (cannabinoid receptors, synthesizing and degradative enzymes) in several brain regions at different phases of the CPP paradigm (expression, extinction, and reinstatement) with morphine. Interestingly, opposite gene expression changes were found between expression and reinstatement phases, whereas no alterations were associated with extinction [138]. 

Other experimental designs were employed to investigate alterations of the ECS after opioid exposure. Vigan et al. analyzed the modifications of AEA and 2-AG levels in the PFC, NAcc, CPu, and Hipp of Sprague–Dawley rats subjected to a morphine behavioral sensitization procedure, divided into three phases (induction, withdrawal, and expression). The results pointed out that the ECS underwent significant changes during the different phases of sensitization to morphine, motivating further studies to address if the functional manipulation of ECS could play a therapeutic role in drug-seeking behavior, particularly in opioid addiction [139]. Besides, the effects of intravenous heroin self-administration on CB1R binding and function were evaluated. There was an enhancement of CB1R density in the VTA and Amy and increased CB1R coupling in the PFC, NAcc, CPu, Hipp, and Amy. These findings suggested that the voluntary chronic intake of opioids regulates CB1R expression and activity in reward-related brain structures, which could represent long-term neuroadaptations, contributing to the development of drug addiction and dependence [140].

Finally, brain endocannabinoid levels (i.e., AEA and 2-AG) were significantly changed in the NAcc, CPu, and mesencephalon of adolescent and adult Long-Evans rats exposed to a postnatal maternal deprivation model (3 h daily from postnatal day 1–14). Interestingly, the vulnerability to morphine reinforcing and motivational actions was significantly higher in deprived rats. Thus, it could be argued that the alterations in brain endocannabinoid levels may be related to the escalation behavior in a self-administration paradigm [141].

#### 3.3.3. Concluding Remarks—ECS and Opioid-Related Disorders

The results compiled suggest a significant involvement of ECS in regulating opioid effects in humans and animal models. However, the available evidence is minimal, and there are many controversies, probably due to differences in experimental designs, particularly in animal model studies. Despite this, it could be proposed that repeated administration of opioids (e.g., heroin, morphine) leads to increased levels and function of CB1R. Better knowledge about the opioid-induced alterations on ECS components and its involvement in the reinforcing and motivational actions of opioids are crucial steps to identify biomarkers with potential usefulness to improve the diagnosis and therapeutics of opioid-related disorders.

### 3.4. Stimulant-Related Disorders

Several studies highlight the functional importance of the ECS in the regulation of the actions produced by psychostimulant drugs, such as amphetamines and cocaine. The importance of studying components of the ECS as a consequence of consumption or exposure to different psychostimulant drugs is fundamental to finding more personalized drugs for the treatment of this type of addiction. In this section, the principal results found in this area are gathered (Table 4).

#### 3.4.1. Gene Polymorphisms of ECS Components

Cocaine-dependent individuals are at high risk of relapsing into heavy use, even after a period of abstinence. There may be a genetic contribution to cocaine dependence and relapse risk. To date, it has been confirmed that few genetic variants contribute to this vulnerability. Two independent *CNR1* variants, the G allele genotypes of rs6454674 (SNP3^G+) and the T/T genotype of rs806368 (SNP8^T/T), have significant interaction effects on the risk of cocaine dependence in European Americans (EA), as well as in African Americans (AA) with these genotypes [142]. Similarly, cocaine-dependent individuals and controls of African ancestry were genotyped for two SNP in the *CNR1* gene (rs6454674, rs806368). A significant difference in genotype frequencies between cases and controls was observed for both SNP [143].

On the other hand, the triplet repeat (AAT)n polymorphism near the *CNR1* gene has been examined in the Afro-Caribbean population among cocaine dependents, with schizophrenia or not. In this case, the frequency of the repeated allele (AAT)12 was increased in both cocaine-dependent non-schizophrenics and schizophrenics compared to the controls [144]. Therefore, all these studies confirm the association between *CNR1* variants and cocaine dependence in different ethnicities.

A variant (rs324420) within the *FAAH* gene on chromosome 1 encodes a substitution (Pro129Thr), which results in decreased FAAH activity, and consequently, increased AEA levels. This has been linked to alterations in mood and stress reactivity and an increased risk of addiction. In a recent study, 70 participants with cocaine use disorder received intravenous doses of saline and cocaine (20, 40 mg). In this study, it was found that the prevalence of the variant allele was higher in the cocaine use disorder group, and also, the drug effect (high and depression) was even higher compared with patients without this polymorphism [145]. In another similar study, this polymorphism was evaluated with methamphetamine (METH)-dependent patients and controls in a Chinese Han population. In these patients, the A allele of the 385C/A (rs324420) polymorphism significantly increased the risk of METH dependence [146].

Similarly, the association of the *FAAH* Pro129Thr polymorphism with METH dependence, METH-induced psychosis, manic episodes, and panic disorder was investigated in a Malaysian population. This polymorphism was significantly associated with the risk of METH dependence and METH-induced mania in this ethnicity [147]. In short, FAAH Pro129Thr polymorphism may contribute to an increased predisposition to cocaine and METH dependence. 

#### 3.4.2. Gene and Protein Function/Expression Changes of ECS Components

METH induces specific damage in the dopaminergic neurotransmitter system and is associated with cell death. A single administration of METH (30 mg/kg, i.p.) alters the concentrations of the endocannabinoids AEA and 2-AG in the striatum of adult male mice, suggesting that the ECS may be involved in brain responses to this drug [148].

Ecstasy, 3,4-methylenedioxymethamphetamine (MDMA), is one of the most widely used psychedelic drugs worldwide. Colado and colleagues revealed that MDMA administration (12.5 mg/kg, i.p.) in adult Dark Agouti rats increased *CNR2* expression, mainly in the microglia of the PFC and hypothalamus [149]. On the other hand, AEA and 2-AG plasma concentrations were not affected by MDMA in a double-blind placebo-controlled study, with 20 healthy recreational polydrug users receiving a pre-treatment with a 5-HT2 receptor blocker (ketanserin, 40 mg) before MDMA (75 mg) [162].

Cocaine is an addictive drug that disrupts mesolimbic dopaminergic neurons by inhibiting dopamine uptake, leading to compulsive behavior and relapse. Acute exposure to cocaine decreased *DAGLA* gene expression and increased tyrosine hydroxylase (TH) in the cerebellum of male C57BL/6J mice. Likewise, chronic cocaine administration decreased FAAH and DAGLβ protein expressions in cerebellar Purkinje cells [150]. Repeated cocaine administration (5 days pretreatment, 20 mg/kg, i.p.) and treatment (10 mg/kg after 6 days of extinction) in the same mouse strain increased *CNR1* gene expression and decreased NAPE-PLD and DAGLα in the hippocampus [151]. However, mice subjected to two sessions of crack cocaine inhalation per day for 11 days reduced mRNA expression levels of ECS components such as *FAAH*, *MGLL* and *CNR1* in the PFC, whereas mRNA levels of the synthesis enzymes *NAPE-PLD* and *DAGLA* were not altered in this brain area [152]. Apparent discrepancies in the results found in these studies may be due to differences in the brain areas (Hipp and PFC), the route of administration (i.p. and inhaled), or the treatment duration (13 and 11 days).

On the other hand, the effects of cocaine sensitization regimen (15 mg/kg, i.p.) for 7 consecutive days during different windows of adolescent vulnerability (PND 33–39, PND 40–46, PND 47–53) on CB1R and CB2R protein levels were evaluated in the PFC and Hipp in male Sprague–Dawley rats. The results revealed that CB1R and CB2R are differentially modulated in PFC and Hipp during adolescence. CB1R protein expression increased, while CB2R decreased in both areas during adolescence compared to adulthood. However, cocaine only altered CB1R and CB2R expression in PND33 to PND39 in the PFC, an effect that did not persist into adulthood, identifying a period of vulnerability during adolescence [153]. Indeed, in a study evaluating CB1R and CB2R status in the cerebral cortex of cocaine-treated adult rodents (mice and rats), CB1R protein was reduced in the PFC. However, CB2R protein was not significantly altered in the PFC of cocaine-dependent rodents [154].

In addition, changes in the expression of CB1R and CB2R in Wistar rats exposed to maternal deprivation (24 h, PND1 and 9) were also evaluated. Overall, maternal deprivation induced sex-dependent and long-lasting alterations in the CB1R expression. Both control and maternally deprived animals received cocaine (8 mg/kg/day) or saline during adolescence (PND 28–48). Adolescent cocaine administration in control animals increased CB1R expression in the Hipp, whereas the opposite was found in maternally deprived animals. Furthermore, cocaine administration during adolescence increased CB1R function in the Hipp exclusively in males in both groups [155]. 

Bystrowska et al. used male Wistar rats to evaluate the changes induced by cocaine after two types of experimental designs: (1) Intravenous self-administration for 14 days, and (2) Intravenous self-administration for 14 days, followed by 10 days of extinction [156]. Immunohistochemical techniques in the brain samples analyzed the levels of CB1R and CB2R. After the self-administration procedure, the results revealed a significant decrease in CB1R expression in the PFC, Amy, and VTA. An increase of CB1R in the Amy was also found in the extinction period. On the other hand, CB2R expression decreased in the PFC and NAcc in any of the methods of cocaine exposure. In another study from the same group, a priming dose of cocaine (10 mg/kg, i.p.) increased the concentrations of AEA in the Hipp and cortex, 2-AG in the Hipp and NAcc, and CB1R and CB2R in the PFC and the lateral septal nuclei only in animals that had self-administered cocaine previously.

Interestingly, drug-induced relapse resulted in an increase in N-Acylphosphatidylethanolamines (NAPEs) levels in cortical areas and striatum and a decrease in oleoylethanolamide (OEA) and palmitoylethanolamide (PEA) levels in the NAcc, cerebellum, and Hipp. Conversely, a down-regulation of CB1R protein expression in the VTA was found. Therefore, all these changes indicate that the ECS is involved in cocaine-reinforced behaviors and drug-induced relapse [157]. 

The results of the ex vivo recordings of neurons in the VTA revealed that cocaine self-administration abolished the mechanisms of ECS-mediated long-term depression (LTD) in glutamatergic synapsis by an altered function of presynaptic CB1R [124]. In another study using the cocaine self-administration paradigm after a prolonged period of abstinence (30 days), increased and reduced DAGL and MAGL levels in the NAcc, respectively, were found during abstinence [123].

In a fascinating comparative study between different rat species, Lewis and Fischer 344 (F344) investigated, displaying a differential sensitivity to cocaine-induced reinstatement, the effects of cocaine self-administration (1 mg/kg per infusion, 21 days) on ECS components expression in the Hipp, which were measured by immunohistochemistry. In this study, Lewis rats showed lower CB1R and higher CB2R expression than Fisher rats measured 24 h after the last self-administration session [160].

Three CB2-specific probes were employed in quantitative real-time PCR (qPCR) and in situ hybridization (ISH) assays to evaluate how cocaine alters CB2R expression in striatal medial spiny neurons expressing dopamine D1 or D2 receptors (D1-MSNs, D2-MSNs) and in microglia. A single injection of cocaine does not alter its expression; however, repeated cocaine injections or self-administration up-regulated CB2R gene expression in both the brain (cortex and striatum) and periphery (spleen). In the striatum, CB2R up-regulation occurred mainly in the D1-MSNs of the NAcc. Brain CB2R could modulate cocaine action through D1-MSNs, considering their significant role in brain reward [161].

Free N-acyl-ethanolamines (NAEs) and 2-acylglycerols levels were analyzed in abstinent cocaine addicts from outpatient treatment programs diagnosed with cocaine use disorder, and in healthy control volunteers. In cocaine use disorder subjects, the NAEs were increased, and 2-acylglycerols decreased compared to controls. In addition, NAEs were significantly elevated in cocaine use disorder patients diagnosed with anxiety compared to cocaine use disorder subjects without this comorbidity. Notably, NAEs concentrations were increased by comorbid mood and anxiety disorders in cocaine addicts. Hence, these results suggest that NAE changes may be biomarkers of cocaine use disorder with psychiatric comorbidity [163]. 

Recently, hair concentrations of glucocorticoids (cortisone, cortisol), 2-AG, AEA, OEA, and PEA were compared among 48 recreational cocaine users, 25 cocaine-dependent users, and 67 non-stimulant controls. Significantly higher hair cortisone concentrations were found in recreational cocaine users and cocaine-dependent users compared to controls. OEA and PEA hair concentrations were substantially lower in cocaine-dependent users than recreational cocaine users and controls. No significant differences in AEA and 2-AG concentrations were found among the studied groups. Based on these results, the HPA axis and ECS appear to be critical regulators for cocaine use disorders [164].

#### 3.4.3. Concluding Remarks—ECS and Stimulant-Related Disorders

Alterations in the different components of the ECS were found at central and peripheral levels in animals and humans exposed to psychostimulant drugs such as amphetamines and cocaine. Specific polymorphisms of the *CNR1* gene or the *FAAH* rs324420 SNP have been associated with the development of dependence on stimulants (i.e., cocaine and METH). Moreover, multiple changes of ECS targets were detected mainly in studies with cocaine, revealing the importance of this system in regulating the different aspects of stimulant addiction, such as reward, motivation, withdrawal, or relapse. Even though more studies in both animals and humans are necessary, the current evidence suggests that ECS components may become promising biomarkers in stimulant-related disorders, and may reach a relevant implication in improving their therapeutic approach.

### 3.5. Tobacco-Related Disorders

According to the World Health Organization (WHO), the tobacco epidemic is one of the biggest public health threats worldwide. Tobacco kills more than 8 million people each year, primarily due to its consumption [165]. In that sense, developing new preventive and therapeutic avenues is crucial to lessen tobacco-related disorders’ impact. Several lines of evidence have shown that the ECS regulates nicotine’s reinforcing and motivational actions, with a particular focus on the therapeutic opportunities related to the pharmacological manipulation of CB1R [166,167]. The close overlap of cannabinoid and nicotinic acetylcholine receptors in reward-related brain areas suggests the interaction between both systems [168] (Table 5).

#### 3.5.1. Gene Polymorphisms of ECS Components

Distinct *CNR1* gene allelic variants have been studied in patients with nicotine dependence. The first one, published in 2008, genotyped 10 SNPs in the *CNR1* in 2 independent samples (Virginia Study of Nicotine Dependence (VAND) and Virginia Study of Anxiety and Neuroticism (VAANX)) to test for association with smoking initiation and nicotine dependence. Concretely, 2 out of 10 markers (SNPs rs6928499 and rs2023239) were significantly associated with both measured parameters. Besides, the authors performed haplotype analyses, determining that the haplotype 1-1-2 of SNPs rs2023239-rs12720071-rs806368 was associated with nicotine dependence in both independent samples. Importantly, all these associations were female-specific according to the stratification by sex [169]. Another study investigated the influence of *CNR1* SNP rs2023239 in nicotine reinforcement, and cue-elicited craving in regular tobacco smokers. The C allele variant of this SNP was associated with reduced nicotine reinforcement, whereas no changes were detected concerning craving measures. These results pointed out the involvement of CB1R in nicotine addiction, although further studies are necessary to improve the understanding of CB1R-dependent mechanisms underlying the regulation of nicotine rewarding properties [170].

In addition, Evans and colleagues evaluated if specific *CNR1* polymorphisms comprising the TAG haplotype (SNPs rs806379, rs1535255, and rs2023239) could be associated with cognitive impairment during nicotine withdrawal. Interestingly, the analyses revealed that tobacco smokers homozygous for the major allele of the *CNR1* SNP rs806379 attenuated the cognitive disruption induced by nicotine withdrawal. This result suggested the therapeutic potential of CB1R blockade for smoking cessation, particularly in individuals with more significant nicotine withdrawal-related cognitive alterations [171]. 

#### 3.5.2. Gene and Protein Function/Expression Changes of ECS Components

Among the first approaches to elucidate the involvement of ECS components on nicotine-induced effects are studies evaluating the consequences of repeated exposure to nicotine in rodents. One of the first reports demonstrates that nicotine produces significant modifications of different ECS components, suggesting its pivotal role in nicotine addiction [172]. Thus, a 7-day administration of nicotine to Wistar rats induced significant changes in the levels of AEA and 2-AG in several brain areas (cerebral cortex, limbic forebrain, striatum, Hipp, and brainstem), as well as concrete alterations of CB1R gene or protein expression in the septum and cerebral cortex, respectively. 

Early-life exposure to nicotine is a critical concern considering the high vulnerability to induce long-term alterations in the normal brain development during infancy and/or adolescence, including those related to disturbances in the modulatory role of the ECS. In this regard, various rodent studies have focused on evaluating how nicotine administration during postnatal or adolescence may impair ECS components’ function or expression. Torres et al. exposed C57BL/6J mouse pups from postnatal day 3 (PND3) to PND14 to tobacco smoke (two 1 h exposures per day, 3R4F reference cigarettes). At different periods (infancy, adolescence, and adulthood), protein expression changes in CB1R, CB2R, NAPE-PLD, DAGL, FAAH, and MAGL in the brainstem and striatum of mice were analyzed. Curiously, differential alterations occurred depending on the brain region and age, although infancy was the most critical period for changes in the ECS components. Authors concluded that exposure to tobacco smoke during critical early life periods could disturb the ECS in regions involved in sudden infant death syndrome, emotional regulation, and vulnerability to drug addiction [173]. 

Likewise, a subchronic nicotine treatment’s short- and long-term effects (0.4 mg/kg/day, i.p.) during adolescence (from PND34 to PND43) on hippocampal and striatal CB1R were analyzed in Wistar rats of both sexes. Interestingly, whereas no short-term changes on CB1R protein expression were observed, there were very significant long-term CB1R density alterations in the hippocampus and striatum, with a similar effect in male and female rats. Furthermore, these modifications were accompanied by a significant down-regulation of mu-opioid receptors in both brain regions and sexes. These results suggested that juvenile nicotine exposure induces profound and long-lasting disturbances in the endocannabinoid and opioid systems, which may account for higher vulnerability to developing drug addiction later in life [174]. In addition, Mateos and colleagues also evaluated the long-term effects of adolescent exposure to nicotine (1.4 mg/kg/day, i.p., from PND28 to PND43) on recognition memory, emotional behavior, and CB1R activity in cingulated cortex and Hipp. Surprisingly, nicotine-induced memory impairments were present only in females, whereas an increase in CB1R function (CP-55,940-stimulated GTPγS binding) was only found in male rats [175].

Consequences of nicotine exposure (7 days, 0.4 mg/kg/day, i.p.) on CB1R density during the adolescence (PND30) or adulthood (PND60) of Sprague–Dawley rats were notably different. In several regions of the PFC and Hipp and the VTA, quantitative autoradiography revealed a significant increase in CB1R binding, while no changes were detected in adult-treated rats. These changes were associated with a higher susceptibility to the locomotor-decreasing effects of both THC and CP-55,940, only in nicotine-treated rats during the adolescent period. These findings confirm the close functional interaction between nicotinic and cannabinoid systems, pointing out that early nicotine exposure could heighten the vulnerability to cannabis use [176]. 

Behavioral sensitization induced by juvenile exposure to nicotine in adolescent Sprague–Dawley rats (PND28) was helpful to classify the animals as low or high responders (depending on the nicotine-induced hyperlocomotion effects). Following a 1-week or 3-weeks injection-free period, all the animals received a low dose nicotine challenge (0.1 mg/kg, s.c.) to evaluate locomotor sensitization, social interaction, and *CNR1* gene expression in the BLA and CeA. Only high responders showed nicotine-induced locomotor sensitization and decreased social interaction, together with a significant reduction of *CNR1* mRNA expression in BLA and CeA after a 1-week injection-free period [177]. 

In another study, nicotine was combined with a high-fat diet (HFD) for 4 weeks to evaluate the consequences on *CNR1* gene expression in hypothalamic nuclei (arcuate, paraventricular, ventromedial, lateral) and Hipp. The authors demonstrated that a significant up-regulation of *CNR1* mRNA levels was found when combining nicotine and HFD. These results suggest enhanced endocannabinoid response in diet-induced obesity combined with nicotine exposure [178].

To date, two publications have specifically evaluated the involvement of the ECS in the regulation of nicotine withdrawal. First, a 7-day nicotine dependence procedure (5.2 mg/kg/day, transdermal patch) was performed in Wistar rats to finally measure several spontaneous somatic withdrawal signs, including locomotor activity and anxiety-like behavior, at 16 h and 34 h after patch removal. AEA levels were significantly lower in the Hipp of abstinent rats at 16 h, whereas they were higher in the Amy and hypothalamus at 34 h of nicotine withdrawal. However, the levels of 2-AG did not differ between nicotine withdrawal and control groups [179]. Second, precipitated nicotine withdrawal was induced in C57BL/6J mice (25 mg/kg/day, 14 days, Alzet minipump), showing episodic memory deficits evaluated in an object recognition task. Interestingly, this cognitive impairment was associated with a significant increase of 2-AG levels and a reduction in MAGL protein expression [180]. Altogether, these findings emphasize how ECS components are significantly modified during nicotine withdrawal, providing essential clues for developing cannabinoid-based pharmacological strategies to maintain abstinence and avoid nicotine relapse.

In a self-administration paradigm, Buczynski et al. compared the effects of volitional intake and forced administration of nicotine on levels of eCBs detected by in vivo microdialysis in the VTA of Wistar rats. Interestingly, nicotine self-administration (volitional), but not yoked administration (forced), reduced oleoylethanolamine (OEA) and increased AEA levels in the VTA in comparison with control animals. However, 2-AG levels increased regardless of the nicotine exposure paradigm, and FAAH activity was not affected. These results demonstrated for the first time that nicotine-induced modulation of eCBs levels was influenced by the volitional nature of drug exposure [181]. 

#### 3.5.3. Neuroimaging of ECS Components

Very few PET imaging studies have linked tobacco consumption and cannabinoid receptors. A PET imaging study using the CB1R selective ligand [^18^F]FMPEP revealed a 20% lower total brain CB1R density in tobacco smokers who were otherwise healthy than the non-smoking group [183]. In a study performed in patients with schizophrenia and healthy controls, tobacco consumption was also studied. The results showed that [^11^C]OMAR *V*_T_ was lower in the Amy, caudate, Hipp, hypothalamus, insula, putamen pallidum, frontal, parietal, occipital, and posterior cingulate cortices of schizophrenic patients than in healthy controls. In contrast, there were no significant differences in [^11^C]OMAR *V*_T_ between smoker schizophrenic patients and healthy controls. Significantly lower [^11^C]OMAR *V*_T_ was detected in non-smoker versus smoker schizophrenics in the parietal and occipital cortices [184]. Interestingly, while in healthy controls, tobacco consumption decreases the availability of CB1R, in patients with schizophrenia, a slight increase was found.

In an earlier study in female rats by Gérard et al., they scanned the rats at baseline and after chronic administration of nicotine using [^18^F]MK-9470. Their results showed that chronic nicotine administration did not significantly change CB1R availability in rats [182].

#### 3.5.4. Concluding Remarks—ECS- and Tobacco-Related Disorders

Although there is limited evidence regarding the role of the ECS components in tobacco addiction, some interesting conclusions can be extracted from the previously reviewed studies. Some polymorphisms of the *CNR1* gene have been suggested to be associated with nicotine dependence, and, interestingly, the rs806379 SNP appears to be a protective factor, since it has been related to an attenuated cognitive disruption during nicotine withdrawal. On the other hand, exposure to nicotine at early life stages is closely related to long-term disturbances in specific ECS markers. Furthermore, animal models evaluating nicotine withdrawal or motivation have revealed differential changes in the levels of eCBs. Finally, very scarce neuroimaging data suggest a reduction of CB1R availability in tobacco smokers. Additional research is essential to improve the understanding of ECS involvement in nicotine addiction and identify potential biomarkers. 

### 3.6. Hallucinogen-Related Disorders

According to the National Institute on Drug Abuse (NIDA), hallucinogens can be classified into two categories: (1) Classical hallucinogens (D-lysergic acid diethylamide (LSD), 4-phosphoryloxy-N,N-dimethyltryptamine (psilocybin), mescaline, N,N-dimethyltryptamine (DMT), and 251-NBOMe), and (2) Dissociative hallucinogens (Phencyclidine (PCP), ketamine, dextromethorphan or Salvia divinorum). Both hallucinogens can generate visual and auditory sensations that are not real. At the same time, dissociative drugs, additionally, can make people who consume these substances feel out of control or disconnected from their body and environment [185]. These substances are primarily used recreationally. Indeed, 7.5% of adolescents consumed hallucinogens at some time in their lives, according to data reported in 2020 [186]. Therefore, due to the potential health impact of hallucinogen use, it is necessary to look for potential biomarkers to design better preventive and therapeutic strategies (Table 6).

#### 3.6.1. Gene and Protein Function/Expression Changes of ECS Components

A study carried out by Guimarães et al. in a cohort of healthy volunteers showed that oral administration of Ayahuasca, a tisane containing alkaloids such as DMT, reduced plasma concentrations of AEA at 4 h, while 2-AG was slightly reduced at one and a half hours, followed by an increase at 4 h [187]. 

On the other hand, several studies evaluated the effect of dissociative drugs on the endogenous cannabinoid system. The subchronic administration of ketamine in mice increased the concentrations of AEA in the CPu, Amy, and PFC. However, 2-AG concentrations were increased in CPu and NAcc, but decreased in PFC. Moreover, *MGLL* mRNA levels were reduced in CPu and PFC, although only MAGL protein expression in CPu was reduced [188]. Acute postnatal administration of PCP in mice reduced CB1R levels in the PFC, while increasing it in the dental gyrus of Hipp [189].

Furthermore, in two sub-chronic studies in rats, it was found that AEA levels were increased in the NAcc after a motor activity test [190] or social interaction [191]. However, in the social interaction study, 2-AG levels increased in the NAcc and CPu, AEA decreased in the medial PFC (mPFC) and Amy, and NAPE-PLD expression increased in the mPFC [191]. Interestingly, Vigano et al. showed that the chronic administration of PCP in rats increased 2-AG levels in PFC, together with an increase in CB1R density in Amy and VTA [192]. 

#### 3.6.2. Concluding Remarks—ECS and Hallucinogen-Related Disorders

Although classical and dissociative hallucinogens produce alterations in the ECS, very scarce information is available, so further studies are needed to analyze their potential use as biomarkers in hallucinogen-related disorders.

## 4. Conclusions and Future Perspectives

The evidence from rodent and human studies gathered in this narrative review highlights the alterations that occur in the main components of the ECS upon exposure to drugs of abuse, especially at early life stages or associated with distinct addictive phases (acute/chronic exposure, dependence, withdrawal, or relapse). It is important to note that the currently available information supports the potential usefulness of identifying changes of cannabinoid receptors, ligands, or enzymes as biomarkers to improve the diagnostic classification of patients with SUD, and increase the success of their pharmacological treatment. Nonetheless, the results included in the present review should be interpreted with caution due to some limitations. For instance, several reviewed studies did not consider differences in gender, age, or associated comorbidities such as polyconsumption, a prevalent circumstance in addicted individuals. Furthermore, there is a great variety in the experimental designs employed, particularly with animal models regarding drug exposure duration, doses, and administration patterns. Thus, one of the biggest challenges in future studies is replicating the available results by applying similar procedures. Only in this way will it be possible to be sure about the direction and magnitude of the changes in the different components of the ECS, which is essential in the search for reliable biomarkers with potential application in the clinical setting. In this regard, a multidisciplinary and translational approach combining cutting-edge technologies (i.e., omics) in biological samples from animal models and patients is crucial to rapidly understanding the complex role that the ECS plays in drug addiction. Finally, well-designed clinical studies employing low invasive methods (e.g., neuroimaging) and accessible biological samples (e.g., blood), as well as specific selection criteria, are mandatory to explore further how ECS components could serve as potential diagnostic, prognostic, monitoring, or therapeutic biomarkers in substance-related disorders.

## Figures and Tables

**Table 1:** Main findings from human and animal studies aimed to identify alterations of ECS components in alcohol-related disorders.

**Alcohol-Related Disorders**					
**Subjects**	**Sample/Intervention**	**Method**	**Measurement**	**Main Outcomes**	**References**
**Gene Polymorphisms**					
Alcoholic dependent patients (n = 137, males) Spanish population	Whole blood	Genotyping	Hare’s Psychopathy Checklist Revised (PCL-R) and TaqIA of the SLC6A3 gene, 3′-UTR microsatellites of CNR1 and C385A FAAH SNP and	Strong positive correlation between PCL-R Factor 1, TaqIA SNP, CNR1 and FAAH genes	[34]
Heavy drinkers (n = 88, males)	Saliva	Genotyping	Craving Visual Analogue Scale for alcohol and 48-bp VNTR of DRD4 and rs2023239 SNP of CNR1	↓ craving in DRD4 long VNTR than in short VNTR	[35]
Healthy adolescents (n = 2087) Mostly European descent	Whole blood	Genotyping	AUDIT questionnaire and 7 SNPs of CNR1, NAPE, FAAH, MGLL and DAGLA	SNPs rs9343525 Cnr1 and rs507961 Mgll strongly correlated with AUDIT score	[36]
Patients with alcoholism (n = 729) and healthy controls (n = 799) Japanese population	Whole blood	Genotyping	5 SNPs of FAAH and 14 SNPs of MGLL	No associations were observed between any of the SNPs and the alcoholism	[37]
Patients with alcoholism (n = 785) and healthy controls (n = 487) Japanese population	Whole blood	Genotyping	Q63R polymorphism of CNR2 gene and DSM IIIR diagnostic criteria for alcohol dependence	The polymorphism was associated with alcoholism	[38]
Patients with CD, OD and/or AD (n = 550) and healthy controls (n = 451) European American population	Whole blood	Genotyping	10 SNPs of CNR1 gene and DSM IIIR or DSM IV diagnostic criteria for drug dependence	SNP3^G and SNP8^T/T were associated with both AD and DD	[39]
Alcohol patients (n = 298; 187 with AD and 111 with AA) and healthy controls (n = 155) Spanish population	Whole blood	Genotyping	3 SNPs of CNR1 gene: rs6454674, rs1049353 and rs806368.	↓ risk with rs6454674-rs1049353-rs806368 haplotype	[40]
Patients with AD (n = 121) and controls (n = 136) Caucasian population	Whole blood	Genotyping	Identification of CNR1 gene 1359 alleles	↑ risk to develop alcohol withdrawal delirium with the homozygous genotype Cnr1 1359A/A	[41]
Patients with AD (n = 196) and control subjects (n = 210) Caucasian population	Whole blood	Genotyping	Identification of CNR1 gene 1359 alleles	No association was found between the polymorphism and alcohol withdrawal-induced seizures	[42]
Alcoholics (n = 10) and matched controls (n = 10)	Postmortem brain tissue (Brodmann areas 9 and 10)	Genotyping	Identification of CNR1 gene rs2023239 SNP	↑ susceptibility to changes in the mesocorticolimbic circuitry involved in AD in individuals with the C allele	[43]
177 subjects Spanish population	Saliva	Genotyping	Screening of CNR1, FAAH, DRD2, ANKK1, COMT and OPRM1 genes polymorphisms	↑ risk of AD in patients with C385A Faah genotype	[44]
Patients with AD (n = 497) and healthy controls (n = 389) American population	Whole blood	Genotyping	Exploration of the involvement of CNR1 rs806368, rs1049353, rs6454674, rs2180619 and FAAH rs324420 SNPs on sleep quality in individuals with AD	C allele carriers (CC/AC) of CNR1 rs6454674 had greater sleep disturbances	[45]
Patients with AD (n = 952) criteria and healthy controls (n = 482) European American and African American population	Whole blood	Genotyping	Identification of FAAH Pro129Thr, rs324420 polymorphism	↑ frequency of Thr129 allele in European American AD population, but not in African Americans	[46]
Heavy drinking youth patients (n = 302)	Whole blood	Genotyping	Identification of FAAH C385A alleles	FAAH AC or AA genotypes were associated with more drinking days and more frequent heavy episodic drinking	[47]
C57BL/6J mice	Knock-in containing the human C385A FAAH SNP	Ethanol binge drinking model	Ethanol consumption	↑ ethanol intake and preference in FAAH A/A mice than in WT FAAH C/C mice	[48]
**Gene and Protein Changes**					
Animal Studies					
**Subjects**	**Sample/Intervention**	**Method**	**Measurement**	**Main Outcomes**	**References**
C57/BJ6 male mice	Two-bottle choice paradigm (2, 4, 8, 16 and 32% of ethanol each 3 days)	qPCR	CB2R gene expression analyses	↑ alcohol preference was associated with ↓ CB2R gene expression in the MB	[38]
Male Wistar rats	3 g/kg of ethanol, p.o., acute	In situ hybridization	Changes on TH, PENK and CB1R gene expressions	↑ TH gene expression in VTA and SN ↑ PENK gene expression in CPu, NAcc C and S, AMY C and M, VMN and PVN ↓ CB1R gene expression in CPu, AMY C and VMN	[49]
Male Wistar rats	10% *v*/*v* ethanol for 52 days, chronic	In situ hybridization	Changes on CB1R gene expression	↓ CB1R gene expression in CPu, VMN and CA1 and CA2 fields of HIPP ↑ CB1R gene expression in the DG	[50]
Male Long-Evans rats	Ethanol vapor exposure 14 h/day, to achieve 150–250 mg/dL BEC for 8–12 weeks	WB and qPCR	Changes on ECS components in the lateral habenula	↑ MGLL and DAGLB, and ↓ CB1R gene expression ↑ MAGL and ↓ CB1R protein levels	[51]
C57BL/6J embryos into host CD1 mothers	ECR1 disruption by CRISPR/CAS9 method + two bottle choice paradigm	qPCR	Changes on CB1R gene expression in different brain regions	↓ CB1R gene expression in the HIPP but not in the hypothalamus, accompanied with reduced ethanol intake	[52]
Wistar female rats and their male pups	MS + forced ethanol consumption (10%)	WB	Evaluation of CB1R and CB2R protein levels	↑ CB1R in the VS and ↓ in the FCx in MS rats = CB2R in all regions in MS rats	[53]
Ethanol-preferring (AA) and non-preferring (ANA) male rats	Ethanol self-administration	In situ hybridization and qPCR	Analyses of ECS components in different brain regions	↓ CB1R protein binding by in-situ hybridization ↓ FAAH and MGLL in the PFC ↓ FAAH protein levels in the PFC	[54]
Male and female Wistar rats	Ethanol vapor exposure (14 h/day, 6 weeks)	qPCR	ECS components in BLA and vmPFC	↓ CNR1, DAGLA and MGLL in the BLA in males ↓ CNR1 in the BLA in females ↓ NAPE-PLD in vmPFC in males	[55]
Male Wistar rats	Chronic or intermittent ethanol treatment (10% *w*/*v*) and withdrawal (at 6 h and 24 h)	qPCR	ECS components in AMY	↓ FAAH gene expression after continuous ethanol exposure and 24 h of withdrawal ↑ NAPE-PLD gene expression after continuous exposure and 6 h of withdrawal ↓ MGLL gene expression after intermittent exposure and 24 h of withdrawal	[56]
Male Wistar rats	Intermittent alcohol exposure	qPCR	ECS components in PFC, ST, AMY and dorsal and ventral HIPP	↓ CNR1 in AMY and ST ↓ CNR2 in HIPP and ST ↑ NAPE-PLD in PFC and ↓ in the AMY	[57]
Clinical Studies					
CA, AS and C patients	Post-mortem brain tissue (Ventral striatum)	WB	CB1R and FAAH enzyme levels	↓ CB1R, FAAH activity and levels in CA	[58]
Alcohol users (7) and non-users (7)	MDDC	qPCR and Flow cytometry	Changes on CB1R, CB2R and GPR55 gene and protein expression levels	↑ CB2R and GPR55 gene expression in MDDC of alcohol users Ethanol treatment increased both target’s gene expression levels	[59]
**Neuroimaging**					
Animal Studies					
Male Wistar rats	Ethanol administration (acute: 4 g/kg, i.p.; chronic: liquid diet containing ethanol 7.2 % *v*/*v*)	PET ([18F]MK-9470)	CB1R binding	↑ CB1R density in the NAcc after acute ethanol ↓ CB1R density in the Hipp and CPu after chronic ethanol, effect reversed after 7 and 14 days of abstinence	[60]
Clinical Studies					
Patients with AD (n = 8) and healthy controls (n = 8)	Scans at least 4 weeks after the last drink	PET ([11C]OMAR)	CB1R binding	↑ CB1R density in the Amy, Hipp, putamen, insula, anterior and posterior cingulate cortices, and orbitofrontal cortex of patients with AD	[61]
In-patients with AD	2 scans: within 3–7 days of admission from ongoing drinking, and after 2–4 weeks of supervised abstinence	PET ([18F]FMPEP-d2)	CB1R binding	1st scan: ↓ CB1R density in patients with AD and negatively correlated with years of alcohol abuse 2nd scan: ↓ CB1R density in patients with AD (similar result as in the 1st scan)	[62]
Healthy social drinkers (n = 20), alcoholic patients (n = 26) and healthy subjects (n = 17)	Acute intravenous ethanol administration vs. chronic heavy drinking	PET ([18F]MK-9470)	CB1R binding	↑ CB1R density after acute intravenous ethanol injection ↓ CB1R density in chronic drinking patients	[63]
Patients with AD (n = 23) and healthy controls (n = 25)	Scans 3–7 days and 2–4 weeks after abstinence	PET ([C-11]CURB)	FAAH binding	↓ FAAH density in alcoholic patients during early abstinence (3–7 days) No changes after 2–4 weeks of abstinence	[64]

AA: alcohol abuse; AD: alcohol dependence; AEA: anandamide; Amy: amygdala (C: central and M: medial); AS: alcohol-dependent suicides; AUDIT: Alcohol Use Disorder Identification Test; BEC: blood ethanol concentration; BLA: basolateral amygdala; C: non-psychiatric controls; CA: alcohol-dependent non-suicides; CB1R/CNR1: cannabinoid receptor 1 (protein/gene); CB2R/CNR2: cannabinoid receptor 2 (protein/gene); CD: cocaine dependence; DAGL: diacylglycerol lipase; DD: drug dependence; FAAH: fatty acid amidohydrolase; FCx: frontal cortex; GC-MS: gas chromatography-mass spectrometry; Hipp: hippocampus (DG: dental gyrus); i.p.: intraperitoneal; LC-MS: Liquid chromatography-mass spectrometry; MAGL/MGLL: monoacylglycerol lipase (protein/gene); MB: midbrain; MDDC: monocyte-derived dendritic cells; MS: maternal separation; NAcc: nucleus accumbens (C: core and S: shell); NAPEPLD: N-Acyl Phosphatidylethanolamine Phospholipase D; OD: opioid dependence; PENK: proenkephalin; PET: positron emission tomography; PFC: medial prefrontal cortex (vmPFC: ventromedial PFC); p.o.: per os; PVN: paraventricular nucleus; qPCR: quantitative real time PCR; s.c.: subcutaneous; SN: substantia nigra; SNP: single nucleotide polymorphism; ST: striatum (VS: ventral striatum); TH: tyrosine hydroxylase; VMN: ventromedial hypothalamic nucleus; VNTR: variable number of tandem repeats; VTA: ventral tegmental area; WB: Western blot; WT: wild type; 2-AG: 2-araquidonoyl glycerol; ↑: increase; ↓: decrease.

**Table 2:** Main findings from human and animal studies aimed to identify alterations of ECS components in cannabis-related disorders.

Cannabinoid Use Disorders					
Subjects	Sample/Intervention	Method	Measurement	Main Outcomes	References
**Gene Polymorphisms**					
Cannabis dependent patients	Whole blood	Genotyping	CNR1 SNP rs1049353	Positive association with CD symptoms ↓ anterior cingulum volume by structural MRI	[67] [68]
			CNR1 SNP rs806380	No association Positive association with CD symptoms	[67] [69,70]
			CNR1 SNP rs806368	Positive association with CD symptoms	[69]
Daily marijuana smokers	Whole blood	Genotyping	CNR1 rs2023239 (allele G)	Positive correlation with significant withdrawal and craving	[71]
	-	fMRI		↑ activity in OFC, IFG, ACG	[72]
Heavy cannabis users	-	fMRI	CNR1 rs2023239 (allele G)	Small hippocampal volume	[73]
Heavy cannabis users	-	fMRI	CNR1 rs1406977	↑ connectivity in the left ventrolateral PFC ↓ working memory	[74]
Schizophrenic patients with CUD	Whole blood	Genotyping	CNR1 SNP rs12720071 (allele G)	Small parietal white matter volume	[75]
			CNR2 rs2501431	An association with the MAPK14 SNP rs12199654 (A-allele carriers) and small cerebral and lobar white matter volumes Positive correlation with CUD	[76] [77]
			CNR2 rs12744386 and rs35761398	High risk for schizophrenia	[78]
Street drug users	Whole blood	Genotyping	FAAH rs324420 (allele A)	Positive correlation with street drug use	[79]
Healthy adult volunteers	-	fMRI		↑ ventral STR reactivity and ↓ AMY reactivity + correlation between STR reactivity and ↑ impulsivity trait − correlation between AMY reactivity and anxiety traits	[80]
Cannabis dependent patients	Whole blood	Genotyping	FAAH rs324420 (allele C)	↑ bias to appetite stimuli	[81]
				↑ risk for CUD	[72,82,83]
				Positive correlation with craving	[71]
		fMRI		↑ activation of OFC, ACG and NAc	[72]
Children with sexual abuse and cannabis dependent symptoms	Whole blood	Genotyping	MGLL SNP rs604300	Positive association with stress adaptation	[84]
**Gene and protein changes**					
Animal studies					
Rats Wistar rats	CP-55,940 (0.4 mg/kg, i.p., 11 days)	In situ hybridization	CB1R mRNA in CPu	↓ CNR1 at 11 days of administration	[85]
	Δ^9^-THC (5 mg/kg, i.p.; 14 days)			↓ CNR1 at 14 days of administration	[86]
Wistar rats	Δ^9^-THC (10 mg/kg, i.p., 5 days)	In situ hybridization	CB1R mRNA in CPu	↑ CNR1	[87]
Long-Evan rats	Δ^9^-THC (1.5 mg/kg, i.p., every third day, from PNDs 28 to 49)	Fluorescent immunosorbent assay	CB1R mRNA in PFC	↓ CNR1	[88]
		LC-MS analysis of endogenous cannabinoids	2-AG levels in PFC	↑ 2-AG	
Swiss Albino mice	Spontaneous CP-55,940 withdrawal (0.5 mg/kg/12 h; i.p.; 6–7 days)	In situ hybridization	Brain CB1R mRNA levels	↑ CNR1 in NAc, VMN, CeA, HIPP (CA1)	[89,90]
C57Bl/6J mice	Spontaneous CP-55,940 withdrawal (0.5 mg/kg/12 h; i.p.; 7 days)	qPCR	CB2R mRNA in NAc	↓ CNR2	[91]
Wistar rats	Δ^9^-THC (5 mg/kg; i.p.; 14 days)	[35S] GTPgammaS binding autoradiography [3H] CB1R-agonist receptor autoradiography	Brain CB1R binding	↓ CB1R binding in striatum, limbic forebrain and cerebellum	[86]
	CP-55,940 (1, 3 and 10 mg/kg; i.p; 2 weeks) and Δ^9^-THC (10 mg/kg; i.p.; 2 weeks)				[92]
	Δ^9^-THC (6.4 mg/kg; i.p.; 7 days)				[93]
NIH Swiss Mice Sprague-Dawley rats	Δ^9^-THC (30 mg/kg; i.p.)				[94]
	Δ^9^-THC (10 mg/kg; i.p.; 21 days)				[95]
Wistar rats	Δ^9^-THC (3 mg/kg; i.p.; 3 days)			↑ CB1R binding cerebellum and HIPP	[96]
Long Evans rats	Δ^9^-THC 1.5 mg/kg; i.p.; PND 28-49)	GC-MS	Brain AEA levels	↑ AEA in NAcc	[88]
Wistar rats	Δ^9^-THC (10 mg/kg; i.p.; 8 days)			↑ AEA in limbic forebrain areas	[97,98]
FAAHC/A mice (C57Bl/6J background)	Acute THC (1 mg/kg)	Immunohistochemistry Immunoelectron microscopy	Analysis of CB1R, GABA and Glu terminals in NAcc and mPFC	↑ CB1R ↑ GABAergic terminals VTA ↑ Glu terminals VTA	[99]
Clinical studies					
THC dependent patients	PBMCs	qPCR and methylation-specific PCR	CB1R mRNA in PBMCs	↑ CNR1 Hypomethylation of CNR1 promoter	[100]
Healthy volunteers	Plasma Acute single THC administration (20 mg/kg)	LC-MS	Endogenous cannabinoids	↓ AEA, 2-AG, PEA and OEA	[101]
Cannabis dependent patients	-	fMRI	FAAH and MGLL	Changes in white matter associated with regional MAGL gene expression (posterior cingulate, parietal cortex, basal ganglia, temporal cortex)	[102]
	Postmortem brain tissue	qPCR			
**Neuroimaging**					
Clinical studies					
Cannabis smokers not seeking treatment (n = 30, males) and control subjects (n = 28, males)	Scans the first day after admission and approximately 4 weeks after abstinence	PET ([18F]FMPEP-d2)	CB1R binding	↓ CB1R density in cannabis smokers CB1R density returned to normal levels after 4 weeks of abstinence from cannabis	[103]
Chronic cannabis users (n = 10) and age-matched healthy subjects (n = 10)	Scan within the first week following the last cannabis consumption	PET ([18F]MK-9470)	CB1R binding	↓ CB1R density in the temporal lobe, anterior and posterior cingulate cortex and NAcc in chronic cannabis users	[104]
Healthy volunteers (n = 14)	THC administration (10 mg, oral, acute) vs. placebo Scans at baseline and long-term after THC exposure	PET ([11C]MePPEP)	CB1R binding	The severity of THC-induced anxiety was directly correlated with the baseline availability of CB1R in the amygdala	[105]
Cannabis dependent patients (n = 11) and healthy controls (n = 19)	Scans at baseline and after 2 and 28 days of monitored abstinence	PET ([11C]OMAR)	CB1R binding	Negative correlation between CB1R availability and withdrawal symptoms after 2 days of abstinence. No significant group differences in CB1R availability in cannabis dependents after 28 days of abstinence	[106]
Chronic, frequent cannabis users (n = 10) and healthy controls (n = 22)	Scans during early abstinence in frequent cannabis users	PET ([11C]CURB)	FAAH binding	↓ FAAH density in cannabis users Lower FAAH was associated with higher trait impulsiveness FAAH binding is dependent on the genetic FAAH polymorphism rs324420 (C385A)	[107]
Cannabis users (n = 14) and healthy controls (n = 18)	Scans after recent cannabis consumption	PET ([11C]CURB)	FAAH binding	FAAH binding was 12% lower in cannabis users. Lower FAAH binding was related to greater use of cannabis throughout the past year	[108]
Cannabis use disorder patients (n = 10, females) and healthy controls (n = 17)	Scans after 3 days of monitored cannabis abstinence	PET ([11C]OMAR)	CB1R binding	↓ CB1R in female patients with cannabis use disorder in specific brain regions (Hipp, Amy, cingulate, and insula). CB1R binding in the Amy was negatively correlated with mood changes (anger/hostility) during abstinence	[109]

ACG: anterior cingulate cortex; AEA: anandamide; Amy: amygdala; CB1R/CNR1: cannabinoid receptor 1 (protein/gene); CB2R/CNR2: cannabinoid receptor 2 (protein/gene); CD: cannabis dependence; CeA: central amygdala; CPu: caudate-putamen; CUD: cannabis use disorder; FAAH: fatty acid amidohydrolase; fMRI: functional magnetic resonance; GABA: gamma aminobutyric acid; GC-MS: gas chromatography-mass spectrometry; Glu: glutamatergic; Hipp: hippocampus; IFG: inferior frontal gyrus; i.p.: intraperitoneal; LC-MS: Liquid chromatography-mass spectrometry; MAGL/MGLL: monoacylglycerol lipase (protein/gene); MAPK14: Mitogen-activated protein kinase 14; NAcc: nucleus accumbens; OEA: oleoylethanolamide; OFC: orbitofrontal cortex; PEA: palmitoyl ethanolamide; PET: positron emission tomography; mPFC: medial prefrontal cortex; PNDs: postnatal days; qPCR: quantitative real time PCR; SNP: single nucleotide polymorphism; STR: striatum; VTA: ventral tegmental area; VMN: ventromedial hypothalamic nucleus; 2-AG: 2-araquidonoyl glycerol; ↑: increase; ↓: decrease.

**Table 3:** Main findings from human and animal studies aimed to identify alterations of ECS components in opioid-related disorders.

Opioid-Related Disorders					
Subjects	Sample/Intervention	Method	Measurement	Main Outcomes	References
**Gene Polymorphisms**					
Former heroin addicts	Whole blood	Genotyping	FAAH SNP 385C>A CNR1 SNP 18087-18131(TAA)_8–17_ CNR1 SNP 1359G>A CNR1 genotype pattern	No association Long repeats (≥14) associated with heroin addiction Protection from heroin addiction with allele 1359A and genotype 1359AA 1359G>A and 6274A>T associated with heroin addiction	[126]
Opiate-dependent outpatients under stable methadone treatment	Whole blood	Genotyping	CNR1 SNP rs2023239	Minor C allele of rs2023239 associated with a protective effect against lifetime MDD	[127]
**Gene and Protein Changes**					
Animal Studies					
Swiss mice	Morphine (8–45 mg/kg, s.c., 5 days)	[3H]-CP55,940 autoradiography [35S] GTPγS binding autoradiography	CB1r binding WIN-55,212-2-stimulated [35S] GTPγS binding	↓ CB1r in the Globus pallidus ↑ CB1r function in the SN and central gray substance	[128]
Wistar rats	Morphine (10–100 mg/kg, i.p., 6 days)	[3H]-CP55,940 autoradiography [35S] GTPγS binding autoradiography In situ hybridization GC-MS	CB1r binding WIN-55,212-2-stimulated [35S] GTPγS binding CB1r mRNA levels AEA levels	↑ CB1r in medial CPu, septum and NAcc ↓ CB1r in the midbrain and cerebral cortex ↑ CB1r function in the cerebral cortex ↓ CB1r function in the brainstem ↓ CNR1 gene expression in the CPu and cerebellum ↑ CNR1 gene expression in the CA2 region of the Hipp and septum No changes	[129] [130] [129] [130]
Sprague-Dawley rats	Morphine (5 mg/kg, s.c., 4.5 days)	[3H]-CP55,940 autoradiography [35S] GTPγS binding autoradiography GC-MS	CB1r binding CP-55,940-stimulated [35S] GTPγS binding AEA and 2-AG levels	↓ CB1r in the cerebellum and Hipp ↓ CB1r function in the NAcc ↓ 2-AG levels in NAcc and Hipp No changes in AEA levels	[131]
Sprague-Dawley rats	Morphine (5 mg/kg, i.p., 5 days)	Proteomics analysis (HPLC-ESI-MS/MS) WB qPCR LC-MS	32 different proteins related to the ECS DAGL, MAGL, and CB2r protein levels MAGL, CB2r, CB1r mRNA levels AEA and 2-AG levels	↓ MAGL levels in the VTA No changes ↓ CNR2 gene expression in the VTA No changes	[132]
Wistar rats	Chronic morphine (10 mg/kg, s.c., 12 days) Acute morphine (10 mg/kg, s.c.)	WB qPCR	CB1r protein levels CB1r mRNA levels	↑ CNR1 protein expression in the cortex, cerebellum and Hipp ↑ CB1R gene expression in the cortex, cerebellum, Hipp, and PBMCs ↓ CB1R gene expression in the cerebellum ↑ CB1R gene expression in PBMCs	[133]
Wistar rats	Acute morphine (5 and 10 mg/kg, s.c.) Chronic morphine (10–40 mg/kg, s.c., 5 days) and morphine challenge (5 and 10 mg/kg, s.c.)	LC-MS	AEA and 2-AG levels	↑ AEA and ↓ 2-AG levels in NAcc (shell) ↑ AEA and ↓ 2-AG levels in NAcc (shell)	[134]
Sprague-Dawley rats	Chronic morphine (10 mg/kg, s.c., 5 conditioning days during CPP)	qPCR	CB2r mRNA levels	↑ CNR2 in the cortex, spleen, and PBMCs ↓ CNR2 in the brainstem	[135]
Sprague-Dawley rats	Morphine withdrawal after chronic exposure (10 mg/kg, s.c., 5 conditioning days during CPP)	WB Immunoelectron microscopy	CB1r protein levels CB1r-positive terminals	↑ CB1r protein expression in the NAcc ↑ CB1r-positive symmetric synapses	[136]
Sprague-Dawley rats	Morphine withdrawal after chronic exposure (10 mg/kg, s.c., 7 conditioning days during CPP)	WB	DAGL and MAGL protein levels	↑ DAGL protein expression in the NAcc No changes in MAGL	[137]
C57BL/6J mice	Morphine (5, 8, 10, and 15 mg/kg; s.c.; 4 conditioning days during CPP)	qPCR	CB1r, CB2r, FAAH MAGL, NAPE-PLD, DAGL mRNA levels in the dorsal Hipp	CPP expression ↑ FAAH and MGLL and ↓ CNR1 and CNR2 gene expression CPP extinction No changes CPP reinstatement ↓ MGLL and ↑ CNR1 gene expression	[138]
Sprague-Dawley rats	Behavioral sensitization to morphine (10, 20 y 40 mg/kg, s.c., 3 days + 5 mg/kg s.c. morphine challenge after 2 weeks of withdrawal)	LC-MS β-counter to measure [14C]ethanolamine	AEA and 2-AG levels FAAH activity	↓ AEA levels in the CPu and Hipp ↑ 2-AG levels in the Hipp No changes	[139]
Lister-Hooded rats	Heroin self-administration (30 µg/kg/inf, i.v., 2-h daily sessions, 16 days)	[3H]-CP55,940 autoradiography [35S] GTPγS binding autoradiography	CB1r binding CP-55,940-stimulated [35S] GTPγS binding	↑ CB1r in the Amy and VTA ↑ CB1r function in the PFC, NAcc, CPu, Hipp, and Amy	[140]
Clinical Studies					
Morphine abusers	Whole blood	qPCR	CB1r and CB2r mRNA levels	↑ CNR1 and CNR2 in PBMCs	[135]

AEA: anandamide; Amy: amygdala; CB1r/CNR1: cannabinoid receptor 1 (protein/gene); CB2r/CNR2: cannabinoid receptor 2 (protein/gene); CPP: conditioned place preference; CPu: caudate-putamen; DAGL: diacylglycerol lipase; FAAH: fatty acid amidohydrolase; GC-MS: gas chromatography-mass spectrometry; Hipp: hippocampus; i.p.: intraperitoneal; LC-MS: Liquid chromatography-mass spectrometry; MAGL/MGLL: monoacylglycerol lipase (protein/gene); MDD: Major Depression Disorder; NAcc: nucleus accumbens; OEA: oleoylethanolamide; PEA: palmitoyl ethanolamide; PFC: prefrontal cortex; qPCR: quantitative real time PCR; s.c.: subcutaneous; SNP: single nucleotide polymorphism; STR: striatum; VTA: ventral tegmental area; WB: Western blot; 2-AG: 2-araquidonoyl glycerol; ↑: increase; ↓: decrease.

**Table 4:** Main findings from human and animal studies aimed to identify alterations of ECS components in stimulant-related disorders.

Stimulant-Related Disorders					
Subjects	Sample/Intervention	Method	Measurement	Main Outcomes	References
**Gene Polymorphisms**					
Cocaine-dependent EA (n = 734) and AA (n = 834) patients	Whole blood	Genotyping	CNR1 SNP rs6454674 rs806368 (allele G)	↑Interaction effects on the risk of cocaine dependence	[142]
Cocaine-dependent AA (n = 926) patients	Whole blood	Genotyping	CNR1 SNP rs6454674 rs806368	Positive association with cocaine dependence	[143]
Cocaine-dependent AC patients with schizophrenia (n = 45)	Whole blood	Genotyping	CNR1 SNP	Positive association with cocaine dependence	[144]
Cocaine-dependent AC patients non-schizophrenic (n = 97)					
Cocaine use disorder patients (n = 70)	Whole blood	Genotyping	FAAH rs324420	Positive association with cocaine use disorder patients ↑Drug effects (high and depression)	[145]
METH-dependent Chinese Han patients (n = 430) and Control (n = 631)	Whole blood	Genotyping	FAAH rs324420 (allele A)	↑Risk of METH dependence	[146]
METH-dependence Malaysian patients	Whole blood	Genotyping	FAAH rs324420	↑Risk of METH dependence	[147]
METH dependence with manic episodes (Total n = 232)				↑Risk of METH-induced mania	
**Gene and protein changes**					
Animal studies					
Adult male mice C57BL/6J	Single high dose of METH 30 mg/kg, i.p	LC-MS	Levels of AEA and 2-AG in STR	↑ Levels of AEA ↓ Levels of 2-AG	[148]
Adult Dark Agouti rats	Acute MDMA administration 12.5 mg/kg	IHC	CB2R mRNA levels	↑Expression of CB2 in microglial cells in de PFC	[149]
Male mice C57BL/6J	Acute cocaine administration 10 mg/kg	qPCR	DAGLα TH in the cerebellum	↓DAGLα ↑TH	[150]
	CS (10 mg/kg) after chronic administration (20 mg/kg)	WB	FAAH DAGLβ in the cerebellum	↓FAAH ↓DAGLβ	
Male mice C57BL/6J	Pre-treatment (20 mg/kg, 5 days) and treatment (10 mg/kg) after 6 days of extinction	qPCR	CB1R, NAPE-PLD, DAGLα mRNA levels in the Hipp	↓CNR1 ↓NAPE-PLD ↓DAGLα	[151]
Male mice C57BL/6J	2 sessions of crack-cocaine inhalation/day for 11 days	qPCR	FAAH, MAGL, CB1R NAPE-PLD and DALGα mRNA levels in the PFC	↓FAAH, MGLL, CNR1	[152]
				Not altered NAPE-PLD and DALGα expression	
Sprague-Dawley rats	CS (15 mg/kg, i.p.) for 7 days (PND 33–39, PND 40–46, PND 47–53)	WB	CB1R and CB2R PFC and Hipp	↑CB1R ↓CB2R PND 33-39 in PFC	[153]
Adult male CD1 mice	Cocaine 20 mg/kg, i.p., 7 days	WB	CB1R Cb2 r in PFC	↓CB1R	[154]
Drug abusers and controls	PFC/BA9 samples			Not alteration in CB2R	
Wistar rats	Cocaine (8 mg/kg/day) in maternally deprived and control animals (PND 28–48)	WB	CB1R in the Hipp	↑CB1R in Control ↓CB1R in Maternally deprived animals	[155]
Wistar rats	Cocaine intravenous self-administration for 14 days	IHC	CB1R, CB2R protein expression in the PFC, Amy, VTA, NAcc and Hipp	↓ CB1R PFC and Amy, ↑CB1R in the VTA ↓CB2R PFC and NAcc	[156]
	Cocaine intravenous self-administration for 14 days after 10 days of extinction			↑CB1R in the Amy ↓CB2R PFC and NAcc	
Wistar rats	Cocaine intravenous self-administration paradigm and Priming of cocaine (10 mg/kg)	Brain sample (Chromatography and IHC)	AEA, 2-AG (Hipp and NAcc) CB1R and CB2R (PFC, LSN, VTA) NAPEs, OEA and PEA (STR, NAcc, cerebellum and Hipp)	↑AEA Hipp—PFC ↑2-AG Hipp—NAcc ↑CB1R and CB2R in the PFC and LSN ↓CB1R in the VTA ↑NAPE in the STR ↓OEA and PEA in the NAcc, cerebellum and Hipp	[157]
Male Sprague-Dawley rats	Cocaine self-administration paradigm 30 days of abstinence	WB	DAGL and MAGL in the NAcc	↑DAGL and ↓MAGL	[158]
Male Sprague-Dawley rats	Cocaine self-administration (0.75 mg/kg/infusion) for 6 to 9 days	Ex vivo electrophysiology in *VTA*	ECS-mediated LTD	Abolished LTD ↓CB1R presynaptic	[159]
Lewis and F344 rats	Cocaine self-administration (1 mg/kg per infusion, 21 days)	IHC	CB1R and CB2R in Hipp	CB1R Lewis rats < CB1R F344 CB2R Lewis rats > CB2R F344	[160]
Naïve WT mice	Single injection of cocaine (10, 20, 30 mg/kg, i.p.)	qPCR ISH	CB2R, D1-MSNs, D2-MSNs in the STR and microglia	No alteration	[161]
	Repeated cocaine administration (10, 20 mg/kg, i.p for 7 days) Cocaine self-administration (1 mg/kg/infusion to 0.5 mg/kg/infusion)			↑CB2R D1-MSNs > D2-MSNs	
Clinical studies					
Healthy recreational polydrug users (n = 20)	Pre-treatment with ketanserin (40 mg), followed 30 min later by MDMA (75 mg)	Chromatography	AEA and 2-AG	No changes	[162]
Abstinent cocaine addicts with and without comorbidities (n = 134)	Plasma	Chromatography	NAEs 2-acylglycerols	↑NAEs ↓2-acylglycerols in Cocaine addicts ↑↑NAE in cocaine addicts with mood and anxiety disorder	[163]
Recreational cocaine users (n = 48), cocaine dependent users (n = 25), and controls (n = 67)	Hair samples	LC-MS/MS	Cortisone, cortisol, 2-AG, AEA, OEA, and PEA	↑Cortisone in recreational cocaine users and cocaine dependent users ↓OEA ↓PEA in cocaine dependent users compared to recreational cocaine users and controls No significant differences AEA and 2-AG levels	[164]

AA: African-American; AEA: anandamide; Amy: amygdala; CB1R/CNR1: cannabinoid receptor 1 (protein/gene); CB2R/CNR2: cannabinoid receptor 2 (protein/gene); DAGLα: Diacylglycerol lipase-alpha; DAGLβ: Diacylglycerol lipase-beta; D1-MSNs: medial spiny neurons expressing dopamine D1 receptors; D2-MSNs: medial spiny neurons expressing dopamine D2 receptors; ECS: endogenous cannabinoid system; EA: European-American; FAAH: fatty acid amidohydrolase; F344: Fisher 344 rats; Hipp: hippocampus; IHC: Immunohistochemistry; i.p.: intraperitoneal; ISH: in situ hybridization; LC-MS: Liquid chromatography-mass spectrometry; LC-MS/MS: liquid chromatography-tandem mass spectrometry; LSN: lateral septal nuclei; MAGL/MGLL: monoacylglycerol lipase (protein/gene); MDD: Major Depression Disorder; MDMA: 3,4-methylenedioxymethamphetamine; METH: methamphetamine; NAcc: nucleus accumbens; NAPE-PLD: N-acyl phosphatidylethanolamine phospholipase D; OEA: oleoylethanolamide; PEA: palmitoyl ethanolamide; PFC: prefrontal cortex (BA9: Brodmann’s area 9); qPCR: quantitative real time PCR; SNP: single nucleotide polymorphism; STR: striatum; VTA: ventral tegmental area; WB: Western blot; 2-AG: 2-araquidonoyl glycerol; ↑: increase; ↓: decrease.

**Table 5:** Main findings from human and animal studies aiming to identify the alterations of ECS components in tobacco-related disorders.

Tobacco-Related Disorders					
Subjects	Sample/Intervention	Method	Measurement	Main Outcomes	References
**Gene Polymorphisms**					
Nicotine dependent patients divided in 2 samples (Virginia Study of Nicotine Dependence (VAND, n = 688) and Virginia Study of Anxiety and Neuroticism (VAANX, n = 961)	Whole blood	Genotyping	CNR1 SNP rs6928499 rs2023239 CNR1 1-1-2 haplotype (SNP rs2023239-rs12720071-rs806368)	Positive association with smoking initiation and nicotine dependence Positive association with nicotine dependence	[169]
Regular tobacco smokers (n = 104)	Whole blood	Genotyping	CNR1 SNP rs2023239 (allele C)	Positive association with reduced nicotine reinforcement	[170]
Tobacco smokers (n = 73)	Whole blood	Genotyping	CNR1 TAG haplotype (SNP rs806379-rs1535255-rs2023239)	Homozygous for the major allele of the CNR1 SNP rs806379 attenuated the cognitive disruption induced by nicotine withdrawal	[171]
**Gene and Protein Changes**					
Animal Studies					
Male wistar rats	Chronic nicotine (1 mg/kg, s.c., 7 days)	[3H]-CP55,940 autoradiography In situ hybridization GC-MS	CB1r binding CB1r mRNA levels AEA and 2-AG levels	↑ CB1r in the cerebral cortex ↓ CNR1 gene expression in the septum AEA levels ↑ in the brainstem and limbic forebrain and ↓ in the Hipp, STR and cerebral cortex AEA levels ↑ in the brainstem and ↓ in the Hipp and cerebral cortex	[172]
Male C57BL/6J mice	Exposure to tobacco smoke from PND3 to PND14 (two 1h exposures per day, 3R4F reference cigarettes)	WB	CB1r, CB2r, NAPE-PLD, DAGL, FAAH, and MAGL protein levels (infancy, adolescence and adulthood)	↓ CB1r, CB2r, NAPE-PLD, FAAH and ↑ DAGL, MAGL in the brainstem during infancy ↓ CB2r and FAAH in the brainstem during adulthood ↓ NAPE-PLD, MAGL and ↑ FAAH in the STR during infancy ↑ FAAH in the STR during adolescence ↓ NAPE-PLD in the STR during adulthood	[173]
Male and female Wistar rats	Subchronic nicotine treatment from PND34 to PND43 (0.4 mg/kg/day, i.p.)	WB	CB1r protein levels (short- and long-term effects)	↑ CB1r in the Hipp of male and female rats (long-term) ↓ CB1r in the STR of male and female rats (long-term)	[174]
Male and female Wistar rats	Chronic nicotine exposure from PND 28 to PND 43 (0.7 mg/kg, s.c.)	[3H]-CP55,940 autoradiography [35S] GTPγS binding autoradiography	CB1r binding CP-55,940-stimulated [35S] GTPγS binding	↓ CB1r in the area 3 of the cingulate cortex of male and female adult Rats ↑ CB1r function in the cingulate cortex (areas 1, 3) and Hipp (CA1, CA3) of male adult rats ↑ CB1r function in the cingulate cortex (area 1) of female adult rats	[175]
Male Sprague-Dawley rats	Nicotine exposure during adolescence (PND30) and adulthood (PND60) (0.4 mg/kg/day, i.p., 7 days)	[3H]-CP55,940 autoradiography	CB1r binding	↑ CB1r in the cingulate cortex (areas 1, 3), frontal cortex (area 2), VTA and Hipp (dental gyrus and CA3) of adolescent rats	[176]
Male Sprague-Dawley rats	Nicotine exposure (0.35 mg/kg, s.c., 4 injections 3 days apart) followed by a nicotine challenge after 1- or 3-week injection-free period (0.1 mg/kg, s.c.)	In situ hybridization	CB1r mRNA levels	↓ CNR1 gene expression in the BLA and CeA of high responder rats	[177]
Male C57BL/6J mice	Nicotine exposure (3 μg/g body weight, i.p., four times/day during 4 weeks) combined with HFD	qPCR	CB1r mRNA levels	↑ CNR1 gene expression in the arcuate, paraventricular, ventromedial and dorsomedial nuclei, lateral hypothalamus and Hipp	[178]
Wistar rats	Nicotine exposure (5.2 mg/kg/day, transdermal patch, 7 days)	LC-MS	AEA levels 2-AG levels	↓ AEA levels in the Hipp during acute nicotine withdrawal (16 h) ↑ AEA levels in the Amy and hypothalamus during protracted nicotine withdrawal (32 h) No changes from control group	[179]
Male C57BL/6J mice	Nicotine exposure (25 mg/kg/day, Alzet minipump, 14 days)	LC-MS WB	AEA and 2-AG levels MAGL and DAGL protein levels	↑ 2-AG levels and no changes in AEA levels in whole brain homogenates ↓ MAGL and no changes in DAGL levels in whole brain homogenates	[180]
Male Wistar rats	Nicotine self-administration (75 mg/kg per Infusion, 0.1 mL per infusion over 4 s)	LC-MS (in vivo microdialysis) LC-MS (brain bulk tissue) Monitoring of d8-arachidonic acid	AEA, 2-AG, OEA and PEA levels AEA, 2-AG, OEA and PEA levels FAAH activity	↑ AEA and 2-AG levels in the VTA ↑ AEA levels in the VTA, SNr, NAcc, dorsal STR, PFC and Amy ↑ OEA levels in the SNr and PFC ↑ PEA levels in the SNr, NAcc, dorsal STR and Hipp No changes	[181]
**Neuroimaging**					
Animal Studies					
Female Wistar rats	Chronic nicotine exposure (1 mg/kg, i.p., 2 weeks)	PET ([18F]MK-9470)	CB1r binding	No major changes	[182]
Clinical studies					
Frequent chronic cigarette smokers (n = 18, men) vs. non-smokers (n = 28, men)	-	PET ([18F]FMPEP-d2)	CB1r binding	↓ CB1r density in tobacco smokers	[183]
Schizophrenic patients smokers (n = 11, men) and non-smokers (n = 14, men) vs. control subjects (n = 18, men)	-	PET ([(11)C]OMAR)	CB1r binding	↓ CB1r density in schizophrenics (smokers and non-smokers) ↓ CB1r density in non-smoker schizophrenics	[184]

AEA: anandamide; Amy: amygdala; CB1r/CNR1: cannabinoid receptor 1 (protein/gene); CB2r/CNR2: cannabinoid receptor 2 (protein/gene); DAGL: diacylglycerol lipase; FAAH: fatty acid amidohydrolase; GC-MS: gas chromatography-mass spectrometry; HFD: high fat diet; Hipp: hippocampus; i.p.: intraperitoneal; LC-MS: Liquid chromatography-mass spectrometry; MAGL/MGLL: monoacylglycerol lipase (protein/gene); NAcc: nucleus accumbens; NAPEPLD: N-Acyl Phosphatidylethanolamine Phospholipase D; OEA: oleoylethanolamide; PEA: palmitoyl ethanolamide; PET: positron emission tomography; PFC: prefrontal cortex; qPCR: quantitative real time PCR; s.c.: subcutaneous; SNP: single nucleotide polymorphism; STR: striatum; VTA: ventral tegmental area; WB: Western blot; 2-AG: 2-araquidonoyl glycerol; ↑: increase; ↓: decrease.

**Table 6:** Main findings from human and animal studies aiming to identify alterations of ECS components in hallucinogen-related disorders.

Hallucinogen-Related Disorders					
Drug	Species	Paradigm	Methods	Findings	Reference
Ayahuasca	Human	Acute (1 mL/kg, p.o.)	LC-MS	↓ AEA Plasma; 2-AG low reduction follow increased plasma levels (in healthy volunteers)	[187]
ketamine	Mice	Subchronic (15 mg/kg, i.p., 7 days)	LC-MS qPCR	CPu: ↑ AEA and 2-AG levels. ↓ NAPE	[188]
				CeA: ↑ AEA levels	
				NAcc: ↑ 2-AG levels	
				PFC: ↑ AEA levels and ↓ 2-AG levels	
		Ketamine hyperlocomotion paradigm	qPCR	CPU & PFC: ↓ mRNA of MAGL	
			Immunoblotting	CPU: ↓ MAGL protein	
PCP	Mice	Acute (post-natal days 7, 9 and 11, s.c.)	Immunohistochemistry	↓ CB1R in the prelimbic mPFC	[189]
				↑ CB1R in the dental gyrus	
	Rat	Sub-chronic (5 mg/kg, 7 days, i.p.)	GC/MS	↑ AEA in the NAcc (after motor activity test)	[190]
		Sub-chronic (twice a day 5 mg/kg 7 days i.p.)	GC/MS	↓ AEA levels in the mPFC and in the Amy but ↑ in NAcc ↑ 2-AG in the NAcc and in the CPu (after social interaction)	[191]
			Immunoblotting	↑ NAPE-PLD expression in mPFC (after social interaction)	
		Chronic-intermittent (2.5 mg/kg, i.p., 4 weeks)	Autoradiographic-binding	↑ CB1R density in Amygdala and VTA; ↓ stimulation in PFC, Hipp, SN, and cerebellum. ↑ stimulation in Globus pallidus.	[192]

AEA: anandamide; Amy: amygdala; CB1R/CNR1: cannabinoid receptor 1 (protein/gene); CB2R/CNR2: cannabinoid receptor 2 (protein/gene); CeA: central amygdala; DAGL: diacylglycerol lipase; FAAH: fatty acid amidohydrolase; GC-MS: gas chromatography-mass spectrometry; HFD: high fat diet; Hipp: hippocampus; i.p.: intraperitoneal; LC-MS: Liquid chromatography-mass spectrometry; MAGL/MGLL: monoacylglycerol lipase (protein/gene); NAcc: nucleus accumbens; NAPEPLD: N-Acyl Phosphatidylethanolamine Phospholipase D; OEA: oleoylethanolamide; PEA: palmitoyl ethanolamide; PET: positron emission tomography; mPFC: medial prefrontal cortex; p.o.: per os; qPCR: quantitative real time PCR; s.c.: subcutaneous; SNP: single nucleotide polymorphism; STR: striatum; VTA: ventral tegmental area; WB: Western blot; 2-AG: 2-araquidonoyl glycerol; ↑: increase; ↓: decrease.

## Data Availability

Not applicable.
